# Ezrin interacts with the tumor suppressor CHL1 and promotes neuronal differentiation of human neuroblastoma

**DOI:** 10.1371/journal.pone.0244069

**Published:** 2020-12-16

**Authors:** Marzia Ognibene, Annalisa Pezzolo

**Affiliations:** 1 U.O.C. Genetica Medica, IRCCS Istituto Giannina Gaslini, Genova, Italy; 2 Laboratorio Cellule Staminali Post Natali e Terapie Cellulari, IRCCS Istituto Giannina Gaslini, Genova, Italy; Duke University School of Medicine, UNITED STATES

## Abstract

In a previous study, we demonstrated that CHL1, the neuronal cell adhesion molecule close homolog of L1, acts as a tumor suppressor in human neuroblastoma (NB), a still highly lethal childhood malignancy, influencing its differentiation and proliferation degree. Here we found that ezrin, one of the ERM (ezrin, radixin, moesin) proteins involved in cytoskeleton organization, strongly interacts with CHL1. The low expression of *EZRIN*, as well as the low expression of *CHL1* and of the neuronal differentiation marker *MAP2*, correlates with poor outcome in NB patients. Knock-down of ezrin in HTLA-230 cell line induces neurite retraction, enhances cell proliferation and migration, and triggers anchorage-independent growth, with effects very similar to those already obtained by CHL1 silencing. Furthermore, lack of ezrin inhibits the expression of MAP2 and of the oncosuppressor molecule p53, whereas it enhances MAPK activation, all typical features of tumor aggressiveness. As already described, CHL1 overexpression in IMR-32 cell line provokes an opposite trend, but the co-silencing of ezrin reduces these effects, confirming the hypothesis that CHL1 acts in close connection with ezrin. Overall, our data show that ezrin reinforces the differentiating and oncosuppressive functions of CHL1, identifying this ERM protein as a new targetable molecule for NB therapy.

## Introduction

Neuroblastoma (NB) is a rare, neural-crest derived pediatric solid tumor. It takes origin from precursor cells of the sympathetic nervous system, preferentially develops in the adrenal medulla, and is likely to spread to other organs [1, 2]. NB patients are classified into low-, intermediate-, and high-risk categories according to the presence of clinical and biological parameters (such as age at diagnosis, *MYCN* amplification, recurrent segmental chromosomal aberrations, diploid DNA index, and poorly differentiated or undifferentiated histology) for therapeutic stratification [3].

We previously described how *CHL1* (close homolog of L1), a neuronal cell adhesion molecule, acts as a tumor suppressor in human NB, leading to cell differentiation and to reduced tumor growth in a preclinical mouse model [4]. CHL1 is a transmembrane receptor belonging to the L1-CAM family. These cell adhesion molecules play an important role in the developing nervous system, providing axon guidance and triggering cell migration [5]. CHL1 is involved in many neurological disorders, representing a crucial molecule in regulating neuronal outgrowth since the first post-natal days [6]. It has been proposed that alterations in genes involved in neurite maturation, such as *CHL1*, can trigger NB itself, inducing high-risk tumor features [7]. Both extracellular and intracellular CHL1 domains interact with many different molecules. More specifically, CHL1 proved to be able to recruit ezrin to the plasma membrane through a specific sequence in its cytoplasmic domain, leading to neurite outgrowth and to branching in cortical embryonic neurons [8]. Ezrin is a filamentous actin-binding protein of the ezrin-radixin-moesin (ERM) family. These proteins are ubiquitous membrane-cytoskeleton linkers and regulators of cellular motility, adhesion, morphology and signaling [9]. ERM proteins mediate cortical neuron growth, morphology and motility *in vitro* [10]. Interestingly, ezrin in particular, seems to play an important role in neuritogenesis [11]. Furthermore, ezrin was found highly expressed in NB cells differentiating into neurons [12].

However, the functional role of CHL1 in NB pathogenesis and its association with other prognostic factors are poorly understood. In this study, we investigated whether ERM proteins interact with CHL1 in NB cells, thus inducing a higher differentiation degree and lower tumor aggressiveness. We identified ezrin as an interesting potential candidate.

## Materials and methods

### Cell cultures

Human certified NB cell lines IMR-32 (ICLC-HTL96021 by ICLC-Interlab Cell Line Collection, IRCCS AOU San Martino-IST, Genova, Italy) and HTLA-230 (kindly provided by E. Bogenmann [13]), were cultured in Dulbecco’s modified Eagle’s medium (DMEM) High Glucose (EuroClone, Milano, Italy) supplemented with 10% FBS (Gibco, ThermoFisher Scientific, Waltham, MA, USA) and with 1% L-glutamine/penicillin-streptomycin. Cells were maintained at 37°C in a humidified atmosphere containing 95% air and 5% CO_2_. The genomic identity of each line was regularly established by array-CGH and cell lines were always tested to certify lack of mycoplasma contamination.

### Plasmids, shRNAs, and transfections

To knock-down ezrin expression, cells were transfected with the silencing Ezrin shRNA lentiviral plasmid (shEzr) (a pool of three target-specific lentiviral vector plasmids by Santa Cruz Biotechnology, Dallas, TX, USA) or with the non-silencing control shRNA plasmid A (shNS) (Santa Cruz Biotechnology). The full-length human *CHL1* gene was sub-cloned into the eukaryotic expression vector pCEFL, as previously described [4]. In order to check whether the expression of the transfected genes persisted over some days, we lysed cells seven days after transfection and carried out Western blot analysis. Transfections were performed using Lipofectamine 2000 (ThermoFisher Scientific) according to the manufacturer’s instructions.

### Immunofluorescence analysis

Immunofluorescence analysis was performed on cells spotted on cytospin slides, that were then fixed with 4% paraformaldehyde for 20 minutes, permeabilized in PBS containing 0.3% Triton X-100 for 5 minutes, and blocked for 30 minutes at room temperature (RT) with 1% BSA in PBS. Primary antibody incubation with the mouse monoclonal anti-ezrin (1:100) (sc-32759; ID: AB_627560 by Santa Cruz Biotechnology) or with the goat polyclonal anti-CHL1 (1:50) (sc-34986; ID: AB_1121563 by Santa Cruz Biotechnology) was performed overnight at 4°C. Secondary antibodies were conjugated to Alexa 488 (green) or Alexa 568 (red) (1:200) (ThermoFisher Scientific) and were used as recommended by the supplier. After washing, the slides were counterstained with 4’,6ʹ-diamidino-2-phenylindole (DAPI) (Vector Laboratories, Peterborough, United Kingdom) and cover-slipped. Samples were imaged by fluorescence microscopy (Axio Imager M2 equipped with ApoTome System, Carl Zeiss, Oberkochen, Germany).

### Western blot analysis and immunoprecipitation assay

Protein lysates were obtained by lysing cells in Staph-A buffer. Proteins (100 μg) were subjected to SDS-PAGE electrophoresis and transferred to PVDF membrane (Millipore), subsequently blots were probed with the primary antibodies, then with the HRP-conjugated secondary antibodies (1:1000) (ThermoFisher Scientific). The anti-human protein primary antibodies were: the mouse monoclonal anti-ezrin (1:200) (sc-32759; ID: AB_627560 by Santa Cruz Biotechnology), anti-moesin (1:100) (sc-13122; ID: AB_627962 by Santa Cruz Biotechnology), anti-p53 (1:100) (sc-126; ID: AB_628082 by Santa Cruz Biotechnology) and anti-MAP2 (1:50) (MS-249-R7; ID: AB_61839 by ThermoFisher Scientific); the goat polyclonal anti-CHL1 (1:200) (sc-34986; ID: AB_1121563 by Santa Cruz Biotechnology); the rabbit polyclonal anti-radixin (1:100) (sc-6408; ID: AB_2253637 by Santa Cruz Biotechnology). The mouse monoclonal anti-β-actin antibody (1:200) (sc-47778; ID: AB_2714189 by Santa Cruz Biotechnology) was used as loading control. Band visualization was performed by ECL Select Western Blotting Detection Reagent (Amersham, GE Healthcare, Little Chalfont, UK), then signal intensity was measured by densitometry using the Image Lab 6.0 software (ChemiDoc, Bio-Rad, Hercules, CA, USA) and was normalized to the loading control. For the immunoprecipitation assay, cells were lysed in a buffer containing Tris-HCl, Triton X-100, and Glycerol. Equivalent amounts of cell proteins (1000 μg) were incubated overnight at 4°C with each anti-ERM protein or anti-CHL1 antibody under constant rotation. Gammabind G-Sepharose (Amersham) (40 μl) was then added and all samples were incubated again under constant rotation for 1 hour and 30 minutes. Immunoprecipitates were washed three times, eluted in Laemmli’s buffer, subjected to SDS-PAGE and transferred to PVDF membrane for Western blotting. (https://dx.doi.org/10.17504/protocols.io.bgecjtaw)

### Kinase activation assay

Cells were starved for 20 hours in a medium containing 0.5% serum before lysis with a buffer containing 20 mM Tris-HCl, 150 mM NaCl, 1 mM EGTA, 1 mM EDTA, 1% Triton X-100, 1 mM β-glycerolphosphate, 1 mM Na_3_VO_4_, 2.5 mM Sodium pyrophosphate, 0.5 mM NaF, 2 mM AEBSF, 20 mg/mL each of aprotinin and leupeptin. Cell lysates were subjected to SDS-PAGE and transferred to PVDF membrane, then blots were incubated with the mouse monoclonal anti-P-p38 (1:1000) (9216; ID: AB_331296 by Cell Signaling Technology Inc., Danvers, MA, USA), or the rabbit polyclonal anti-P-ERK 1/2 (1:1000) (9101; ID: AB_331646 by Cell Signaling Technology). To check the total amount of loaded proteins, the blots were re-probed with the rabbit polyclonal antibodies against p38 (1:100) (sc-535; ID: AB_632138 by Santa Cruz Biotechnology) and ERK 2 (1:100) (sc-154; ID: AB_2141292 by Santa Cruz Biotechnology).

### Cell growth and morphology

To determine growth rate, 5×10^4^ cells were plated in 12-well plastic plates 48 hours after transfection and were cultured for 24, 48, 72, and 96 hours. At each harvest point, viable cells were trypsinized and counted in Trypan blue. For morphological observations, cells cultured for 72 hours were previously photographed under the CKX41 phase-contrast microscope Olympus (Tokyo, Japan) equipped with the Altra-20 digital camera and with the AnalySIS getIT imaging acquisition software (Olympus). Neurite-like elongation in NB cells was analyzed for the presence of protrusions longer than one cell diameter and the percentages of neurite-extending cells were calculated.

### Cell migration assay

2×10^5^ cells were collected 48 hours after transfection, resuspended in 100 μl of serum-free medium, and plated in the upper insert (8.0 μm pore size) of a Transwell chamber (Corning Costar, Cambridge, MA), with 600 μl of complete medium filling the lower chamber. After 24 hours at 37°C, migrated cells were detached from the underside of the upper insert by 5 mM EDTA and counted in Trypan blue. (https://dx.doi.org/10.17504/protocols.io.bgd9js96)

### Anchorage-independent growth assay

2×10^4^ cells were collected 48 hours after transfection, suspended in DMEM with 10% FBS and 0.3% agarose (SeaKem ME by Lonza, Basel, Switzerland), and plated on top of a 0.6% agarose layer in p60 plastic plates. Colonies grown in soft agar were scored after 10 days and photographed under a phase-contrast microscope (Olympus). (https://dx.doi.org/10.17504/protocols.io.bgeajtae)

### Statistical analysis

Experiments were performed at least three times. For all analyses, the significance of differences between experimental samples and controls was determined by ANOVA analysis with Bonferroni Multiple comparison Test (* *p* < 0.05; ** *p* < 0.01; *** *p* < 0.001). The microarray data generated from the R2 Genomic Analysis and Visualization Platform were examined using the public R2 program for analysis and visualization of microarray data (http://r2.amc.nl) [14]. Event-free survival and overall survival were evaluated by the Kaplan Meier method, splitting NB patients into two groups based on the median value of each gene expression profile. We performed on-line analyses and evaluation of *CHL1*, *EZRIN* and *MAP2* gene expression comparing patient subgroups in different NB datasets downloading the resulting survival curves, box plots, and *p*-values (calculated with log-rank test).

## Results

### High levels of *EZRIN* and *MAP2* expression correlate with better NB patients’ outcome

**Fig 1:**
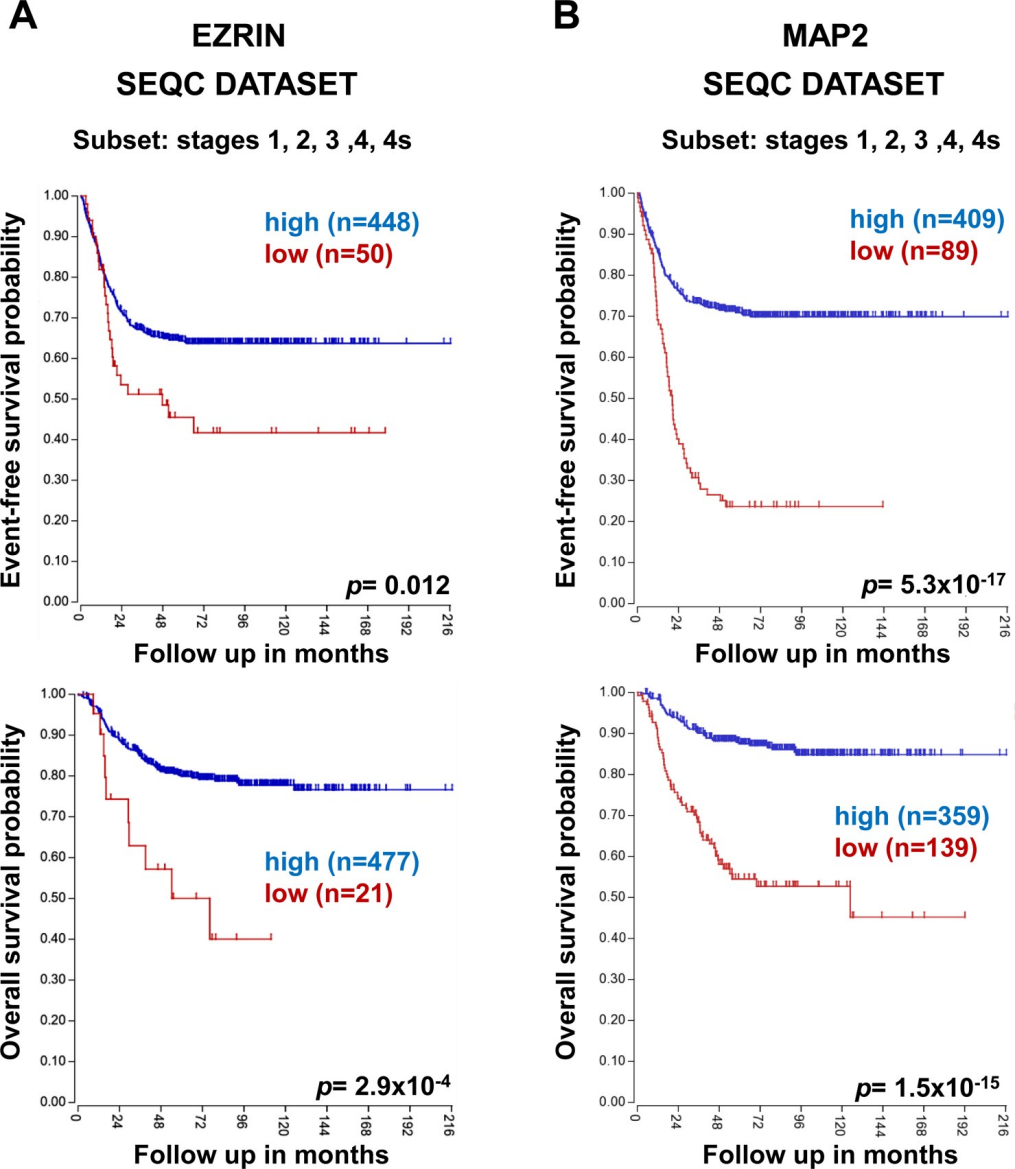
NB patients’ outcome based on *EZRIN* and *MAP2* gene expression. Using the SEQC patients’ datasets in the R2 Genomics Analysis and Visualization Platform (http://r2.amc.nl), event-free survival (top) and overall survival (bottom) curves were generated for **(A)** *EZRIN* and **(B)** *MAP2* (Microtubule-associated protein-2) expression in all NB stages patients. Patients’ numbers (n) are shown in parentheses.

We investigated the correlation between *EZRIN* gene expression and NB patients’ survival, considering all the disease stages defined by the International NB Staging System (INSS) [15], consulting SEQC dataset [16] from R2 Genomics Analysis and Visualization Platform (http://r2.amc.nl). Similarly, we analyzed gene expression of the neuronal differentiation marker *MAP2* (Microtubule-associated protein-2) [17], whose *in vitro* protein levels we had already observed to be directly proportional to CHL1 expression [4]. We found that both *EZRIN* and *MAP2* higher expression levels were significantly associated with higher event-free and overall survival rates in NB patients (Fig 1A and 1B), which suggested a role of these molecules together with CHL1, in leading to a more favorable NB outcome by inducing cell differentiation.

### Ezrin and CHL1 proteins localize to cell surface and strongly interact in NB cells

**Fig 2:**
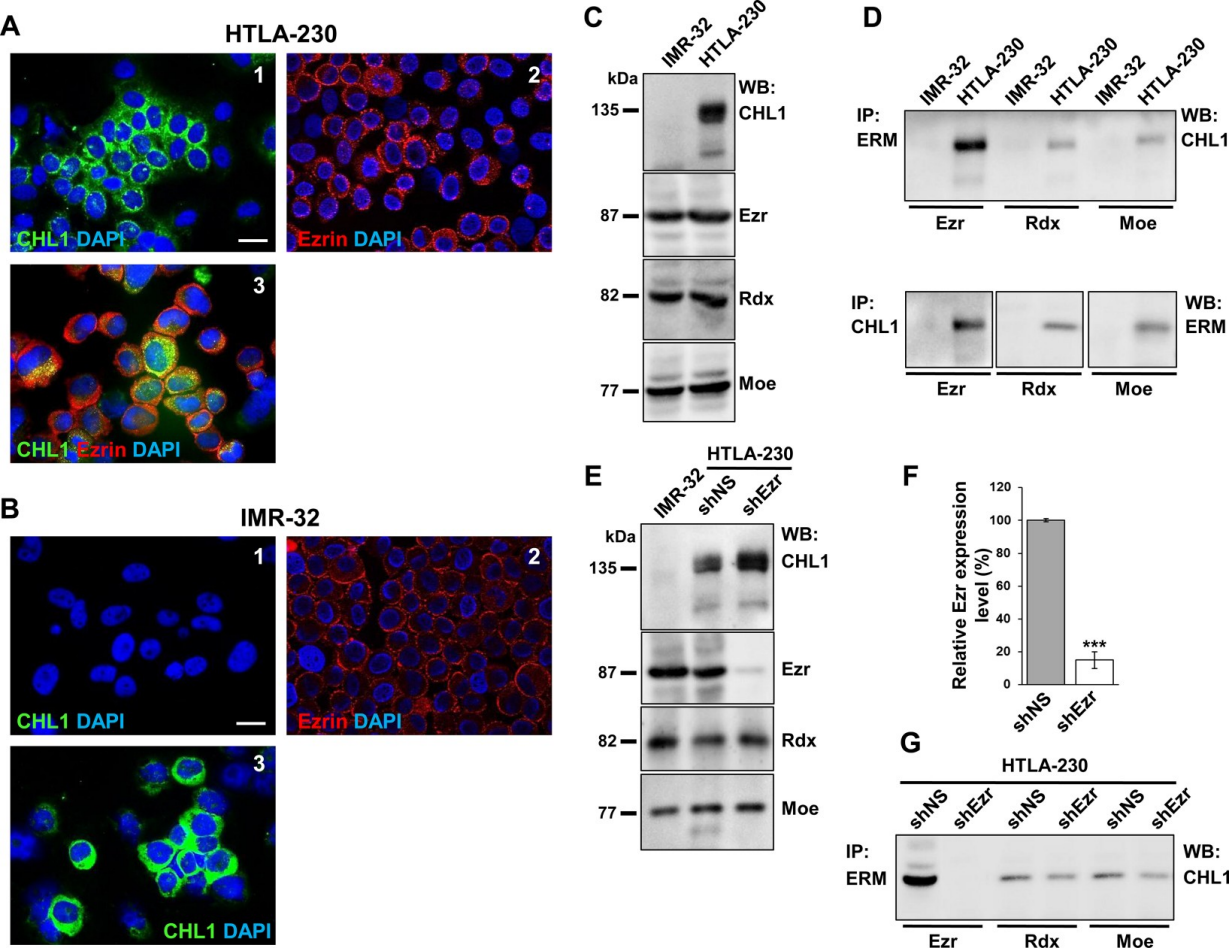
Ezrin-CHL1 localization and interaction in NB cells. Immunofluorescence analysis of CHL1 and ezrin plasma membrane localization on **(A)** HTLA-230 and **(B)** IMR-32 cells using anti-CHL1 (green) (1) or anti-ezrin (red) (2) antibodies. HTLA-230 in photo A3 were double stained for CHL1 and ezrin (yellow, merge). IMR-32 in photo B3 expressed the transfected pCEFL-CHL1 molecule. Cells were counterstained with DAPI to visualize nuclei (blue) (Scale bars: 10 μm) **(C)** IMR-32 and HTLA-230 cells were lysed and subjected to Western blot (WB) analysis with anti-CHL1, anti-ezrin (Ezr), anti-radixin (Rdx), and anti-moesin (Moe) antibodies. Only HTLA-230 cell line displayed expression of endogenous CHL1 protein, whereas the three ERM proteins (Ezr, Rdx, and Moe) were equally expressed in both cell lines, and each of them was used as a loading control. **(D)** Cells lysates were subjected to each anti-ERM protein immunoprecipitation (IP) followed by anti-CHL1 Western blot or to anti-CHL1 immunoprecipitation followed by each anti-ERM protein Western blot. **(E-F)** Cell lysates from IMR-32 (as CHL1 negative control), and from HTLA-230 transfected with shNS (as non-silencing control) or shEzr, underwent Western blot analysis with anti-CHL1 and each anti-ERM protein antibody. HTLA-230 cells were lysed 7 days after transfection. 85–90% reduction in total ezrin levels was achieved compared to the non-silenced control, considered as 100%, whereas radixin and moesin levels did not change and they were used as loading controls. Data are representative of three independent experiments ± SD (*** *p* < 0.001). **(G)** Cell lysates from HTLA-230 transfected with shNS or shEzr were subjected to each anti-ERM protein immunoprecipitation followed by anti-CHL1 Western blot. All the immunoprecipitation results are representative of three independent experiments.

To analyze ezrin and CHL1 protein expression, we chose two NB cell lines, HTLA-230 and IMR-32, both characterized by amplified *MYCN* as a marker of higher NB malignant potential [18]. Differently from IMR-32, HTLA-230 had good expression of endogenous CHL1 protein. We checked cellular localization of ezrin and CHL1 proteins in both cell lines by immunofluorescence staining, observing their co-localization at the plasmamembrane in HTLA-230 cells, as expected. For the IMR-32 cell line, we analyzed cells transfected with pCEFL-CHL1 plasmid (as described below in the text) showing that the transfected molecule appeared well expressed and had the same localization as that of the wild type protein (Fig 2A and 2B).

We tested the expression rate of each of the three ERM proteins in Western blot, and we found that ezrin, radixin, and moesin were equally expressed in the two cell lines (Fig 2C). To assess the ability of CHL1 in binding ERM proteins, cell lysates from untransfected HTLA-230 and IMR-32 cells were subjected to immunoprecipitation for each ERM protein, followed by CHL1 Western blot and vice versa (Fig 2D). CHL1 formed a complex with each of the ERM proteins, showing a stronger linkage with ezrin in both immunoprecipitation assays. Therefore, we proceeded in testing ezrin as a possible enhancer of CHL1 oncosuppressive functions. We knocked-down ezrin in HTLA-230 cell line by transfecting cells with a pool of three lentiviral small hairpin RNA (shRNA) plasmids (shEzr), achieving 85–90% expression reduction compared to the non-silenced control (shNS). Radixin and moesin were not affected by ezrin silencing, which proved the high specificity of the shRNA plasmids used (Fig 2E and 2F). When we performed the immunoprecipitation assay on transfected HTLA-230 cells, we observed that the residual amount of ezrin still expressed by shEzr-cells did not induce any protein complex with CHL1, and we confirmed that the linkage between CHL1 and ezrin was the strongest one among ERMs (Fig 2G).

### Ezrin knock-down interferes with differentiation of NB cells and enhances proliferation

**Fig 3:**
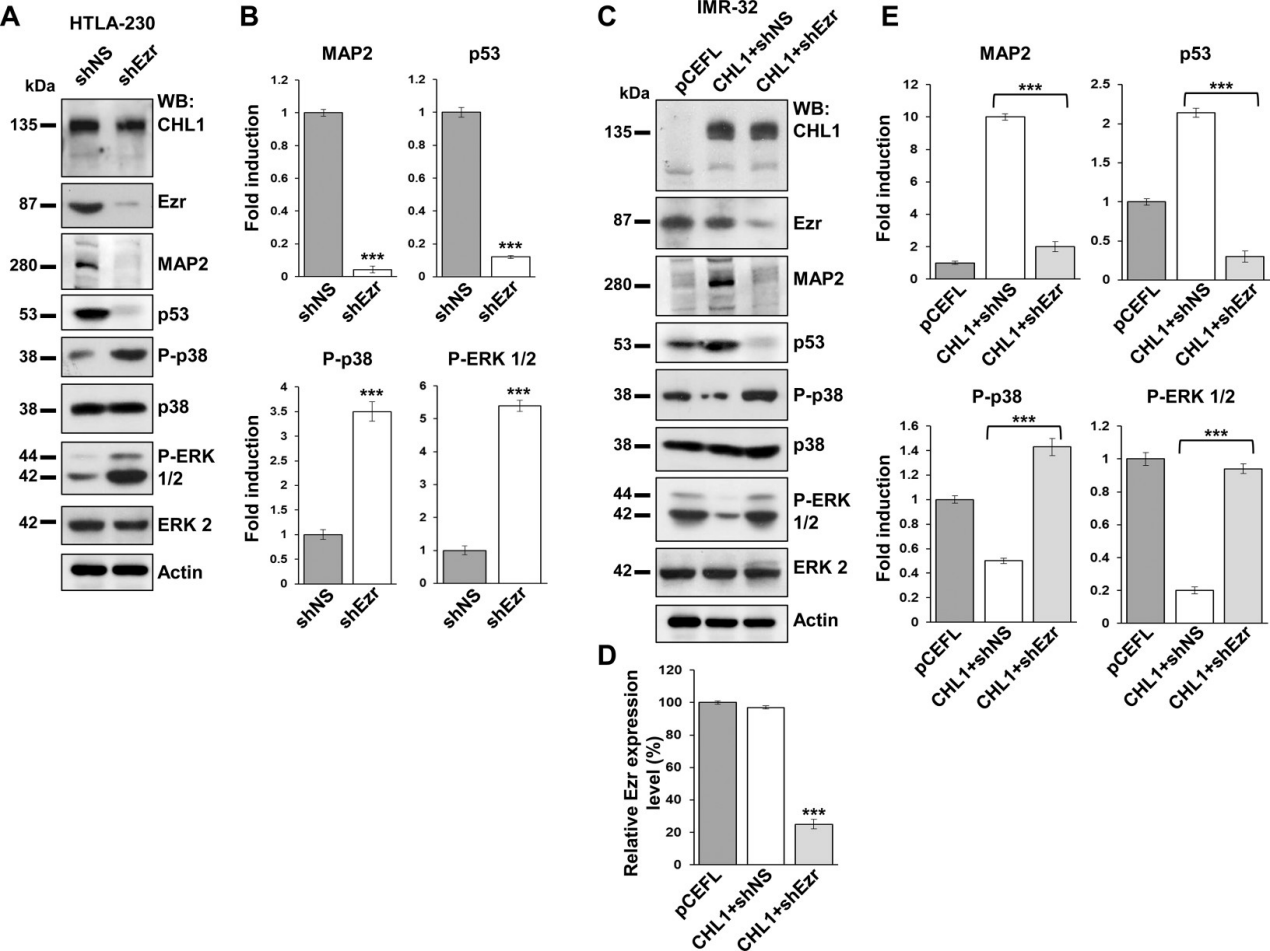
Ezrin silencing in NB cells affected the expression of MAP2, p53, and MAPKs. **(A-C)** Proteins from HTLA-230 cells transfected with shNS (control) or shEzr and from IMR-32 cells transfected with the empty vector pCEFL (control) or cotransfected with pCEFL-CHL1 and shNS or shEzr, were lysed 7 days after transfection, subjected to Western blot, and probed with anti-CHL1, anti-ezrin, anti-MAP2, anti-p53, anti-phospho-p38 (P-p38), and anti-phospho-ERK 1/2 (P-ERK 1/2) antibodies. **(D)** 75–80% ezrin expression reduction was achieved in shEzr-IMR-32 cells compared to the non-silenced controls (pCEFL was considered as 100%) with actin used as the loading control. **(B-E)** Protein levels were quantified by densitometry, normalized to the control values (fold induction = 1) and to the contents of the loading control proteins (actin or total p38 and ERK 1/2), and then visualized by histograms. Data are representative of three independent experiments ± SD. (***p < 0.001).

We assayed MAP2 expression in HTLA-230 cells transfected with shEzr. MAP2 almost disappeared after silencing of ezrin (Fig 3A and 3B), which suggested a lower degree of differentiation. Loss of ezrin also decreased the expression of the oncosuppressor protein p53 [19] pushing cells to proliferate (Fig 3A and 3B). Furthermore, ezrin silencing affected the signaling pathways of the mitogen-activated protein kinases (MAPKs) p38 and ERK 1/2, that resulted about 3.5 and 5 fold more activated than the control, inducing cell migration and transformation, respectively [20, 21] (Fig 3A and 3B). To prove that CHL1 and ezrin strictly cooperate in inducing NB cell differentiation, we overexpressed CHL1 in IMR-32 cells by transfecting them with pCEFL-CHL1, or with the empty vector pCEFL as negative control, as already done in our previous work [4], and we cotransfected shEzr or the negative silencing control shNS. We obtained a strong expression of CHL1 in pCEFL-CHL1-transfected cells, and 75–80% ezrin silencing in CHL1-shEzr-transfected cells (Fig 3C and 3D). MAP2 and p53 expression sharply augmented with CHL1 over-expression and almost disappeared with silenced ezrin (Fig 3C and 3E). MAPKs p38 and ERK 1/2 were respectively about 53% and 80% less activated than control pCEFL-transfected-cells in CHL1-transfected cells, whereas they both resulted strongly re-activated if ezrin was down-modulated (Fig 3C and 3E).

**Fig 4:**
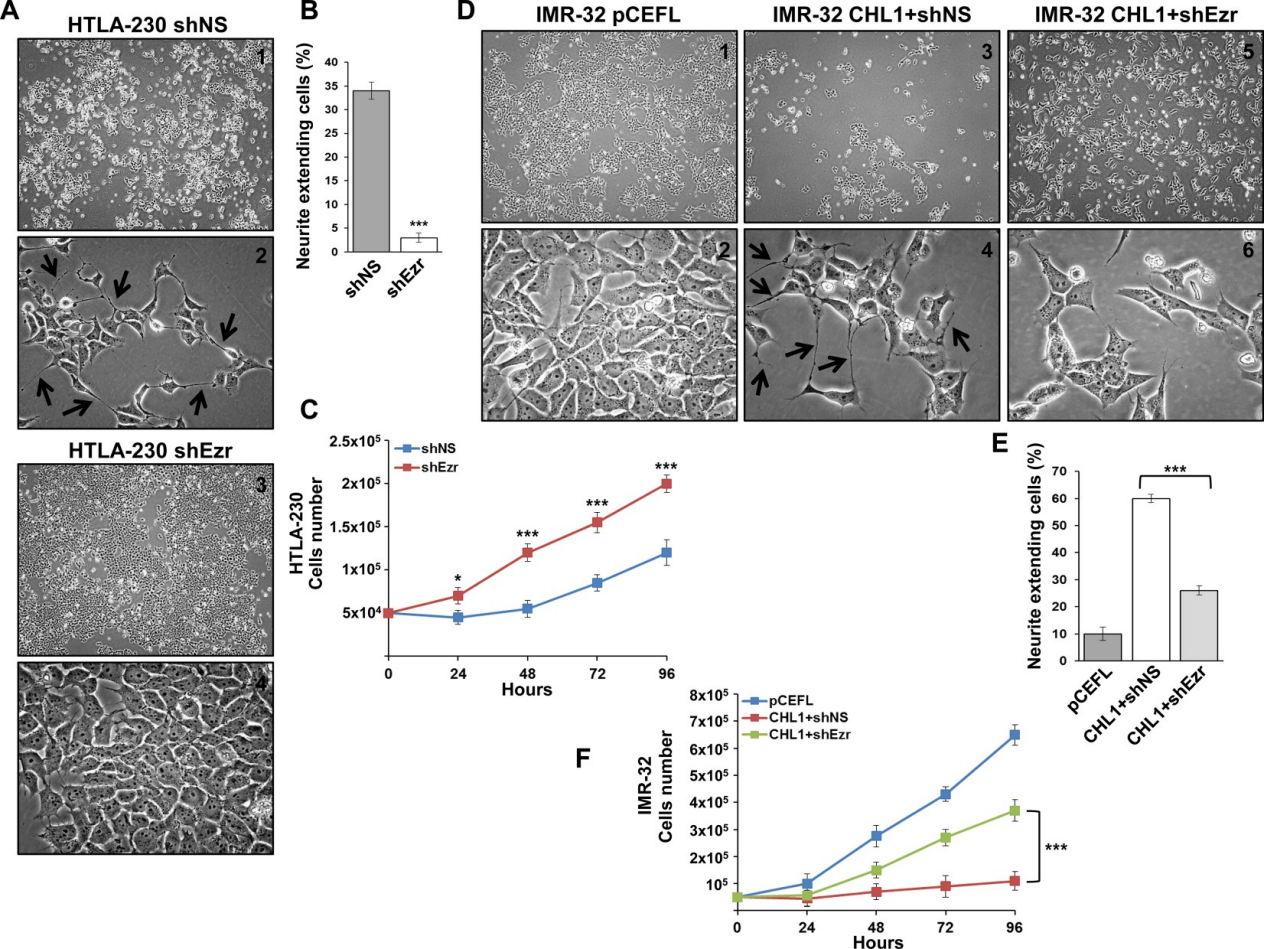
Ezrin knockdown interferes with neuronal differentiation and enhances proliferation in NB cells. Morphological characteristics of cells plated 48 hours after transfection and photographed three days later. **(A)** HTLA-230 cells transfected with shNS (1–2) or shEzr (3–4): shEzr-HTLA-230 grew faster and lost the neurite-like extensions detectable in control cells (see arrows in photo 2). **(D)** IMR-32 cells transfected with the negative control pCEFL (1–2) or cotransfected with pCEFL-CHL1 and shNS (3–4) or shEzr (5–6): over-expression of CHL1 in IMR-32 cells strongly reduced growth rate and induced neural differentiation with protrusion of neurite-like extensions (see arrows in photo 4), whereas the co-silencing of ezrin clearly reduced CHL1 effects. (Magnification 4x and 40x). **(B-E)** Histograms report the percentages of cells extending neurite-like protrusions. Data are representative of three independent experiments ± SD (*** *p* <0.001). **(C-F)** HTLA-230 or IMR-32 cells were plated 48 hours after transfection or cotransfection and cultured for 4 days. Every day, cells were trypsinized and counted. Data are representative of three independent experiments ± S.D. (* *p* < 0.05; *** *p* < 0.001).

These results were confirmed by the observation of cell morphology and growth rate. HTLA-230 cells transfected with shEzr were no longer able to produce neurite-like extensions (Fig 4A and 4B) and, after 4 days, they showed a proliferation rate approximately 2-fold higher than in the control (Fig 4C). CHL1 over-expression induced IMR-32 cells to form many neurite-like protrusions as a sign of a higher differentiation degree, with a growth rate 6-fold lower than in the control; when IMR-32 were cotransfected with shEzr, they gradually lost the differentiated morphology and restarted to grow faster (Fig 4D–4F).

### Ezrin silencing increases invasiveness of NB cells

**Fig 5:**
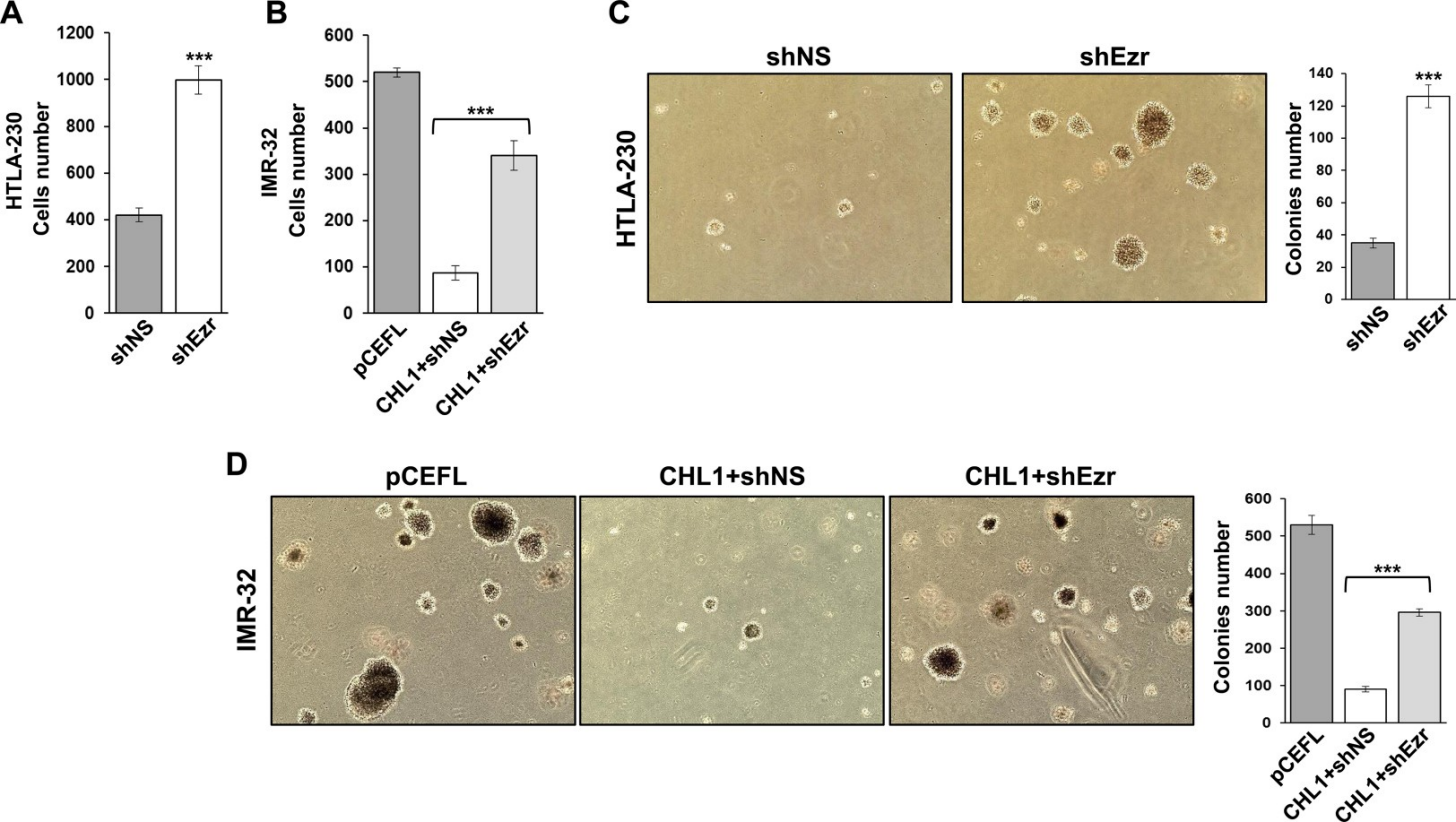
Ezrin down-regulation in NB cells increases migration and anchorage-independent growth. A migration assay was conducted for **(A)** HTLA-230 cells transfected with shNS or shEzr and for **(B)** IMR-32 cells transfected with pCEFL or cotransfected with pCEFL-CHL1 and shNS or shEzr. Cells were seeded 48 hours after transfection in the upper chamber of a Transwell insert: the ones which migrated to the lower side 24 hours later were detached and counted. (Three independent experiments ± S.D). **(C)** Colonies formed by HTLA-230 cells transfected with shNS or shEzr and by **(D)** IMR-32 cells transfected with pCEFL or cotransfected with pCEFL-CHL1 and shNS or shEzr. Cells were collected 48 hours after transfection and cultured in soft agar for 10 days. (Magnification 4x). Histograms show the mean colony number from three independent experiments ± S.D. (***p < 0.001).

To determine whether the loss of differentiation, induced by ezrin down-regulation, affected the invasiveness potentiality of NB cells, we evaluated their migration rate through a Transwell chamber and their ability to grow in an anchorage-independent manner. Both shEzr-HTLA-230 cells and CHL1-IMR-32 cells cotransfected with shEzr had a higher migration rate compared to their relative controls (Fig 5A and 5B). In a soft agar assay, we found that silenced ezrin induced HTLA-230 cells to form larger colonies, and 3.5-fold more numerous than controls (Fig 5C), similarly to what happened in shEzr-IMR-32 cells, where CHL1 over-expression effects proved to be completely reversed (Fig 5D). In summary, our data showed that the lack of ezrin gave results very similar to the ones obtained *in vitro* by CHL1 silencing [4], and that CHL1 and ezrin strictly interact and are mutually essential in inducing NB cell differentiation and a reduced potential of proliferation, migration and transformation.

### High *CHL1* expression is associated with a more favorable NB outcome

We evaluated *CHL1* gene expression in association with known clinical and biological parameters, predictors of NB patients’ survival, using the R2 Genomics Analysis and Visualization Platform and considering data from four independent NB patients’ datasets, namely SEQC [16], Versteeg [14], Lastowska, and Maris [22].

In NB patient cohorts separated by tumor stage [15] from two different datasets, we found that a more expressed *CHL1* gene correlated with better event-free survival rates in patients with both low stage (1, 2, 3, 4S) and high stage (4) disease (S1A Fig). Furthermore, *CHL1* gene expression was significantly lower in patients with stage 4 compared to other stages (S1B Fig). In cohorts separated by age from Versteeg dataset, low *CHL1* gene expression was associated with lower relapse-free and overall survival rates in patients both younger and older than 18 months at diagnosis (S2A Fig). Using the SEQC dataset, we separated NB patients by the presence or the absence of *MYCN* amplification, a well-known condition increasing NB malignant potential [18]. Patients with high *CHL1* gene expression had higher rates of event-free and overall survival in both clusters with *MYCN* non-amplified and amplified tumors, even if statistical significance was not reached for the *MYCN*-amplified group (S2B Fig). In order to establish whether *CHL1* gene expression was related to cytogenetic markers of grim prognosis recurrently observed in NB (chromosome 1p deletion, 11q deletion, and 17q gain) [23], we analyzed microarray expression results from two independent datasets, Lastowska and Maris, that provide information about tumor 1p, 11q, and 17q status. We found that *CHL1* gene expression was weakly lower in tumors from patients with chromosome 1p deletion, 11q deletion, and 17q gain than in the ones lacking these aberrations (S3 Fig). Therefore, *CHL1* seems to have minimal correlation with the most important NB segmental chromosomal alterations, similarly to what we observe for the *EZRIN* gene too (S4 Fig). Nevertheless, our results generally confirmed the association of high *CHL1* gene expression with more favorable outcome in all NB patients’ subsets, validating its oncosuppressive properties [4].

## Discussion

Here we provide evidence that CHL1 firmly interacts with ezrin in NB cells, and that this interaction leads to a higher neuronal differentiation degree and, consequently, to a lower tumor invasiveness potential. We also found that the low expression of *EZRIN* and of the neuronal differentiation marker *MAP2* was related to poor prognosis and lower survival rates for NB patients. Moreover, we report statistical data supporting the unambiguous influence of *CHL1* expression on most NB-determining clinical factors.

We previously demonstrated that *CHL1* acts as a tumor suppressor gene in NB and that its over-expression causes cell differentiation and clearly reduces proliferation and tumor growth in a preclinical mouse model [4]. Subsequent studies found CHL1 preferentially expressed in low-risk NB [24]. Consulting public NB patients’ datasets, we remarked that high *CHL1* expression was related to better outcomes and higher survival rates, considering tumor stage, age at diagnosis, and *MYCN* oncogene amplification.

It is known that L1 molecule binds to the ERM family members to coordinate axon morphogenesis in hippocampal neurons [25] and that CHL1 can link ERM proteins, more specifically ezrin, to mediate neurite development [8]. Furthermore, since activated ERMs play a significant role in neural tissue growth [10], we investigated CHL1-ERM interaction in NB cells and whether this interaction can enhance neuronal differentiation degree. We started by consulting public NB patients’ datasets and we found that the higher expression of *EZRIN* and *MAP2* genes was associated with good outcome of NB patients. MAP2 is a neuronal differentiation marker and an essential molecule for the correct development of neuron morphology [17]. By immunoprecipitation assay, we evaluated CHL1 interaction with each of the ERM proteins, finding that the most stable and strong protein complex was obtained with ezrin. Knock-down of ezrin in HTLA-230 cells produced a marked loss of differentiation in cell morphology, associated with higher proliferation and migration induced by enhanced levels of activated ERK 1/2 and p38 MAPKs, respectively. As expected, protein expression of MAP2 and p53 was inhibited, and cells acquired the ability to form more numerous and larger colonies in an anchorage-independent condition, a typical feature of tumor invasiveness. These results were similar to the ones already obtained by silencing *CHL1* gene [4], suggesting a comparable role of ezrin in NB cell physiology. We used IMR-32 cells to overexpress CHL1, barely expressed in this cell line, and to simultaneously silence ezrin. We observed that the higher differentiation degree and the reduced proliferation and migration abilities conferred by CHL1 overexpression were reverted by the lack of ezrin. This result revealed a strict connection between the molecular functions of CHL1 and ezrin in NB cells, with particular regard to differentiation induction.

**Fig 6:**
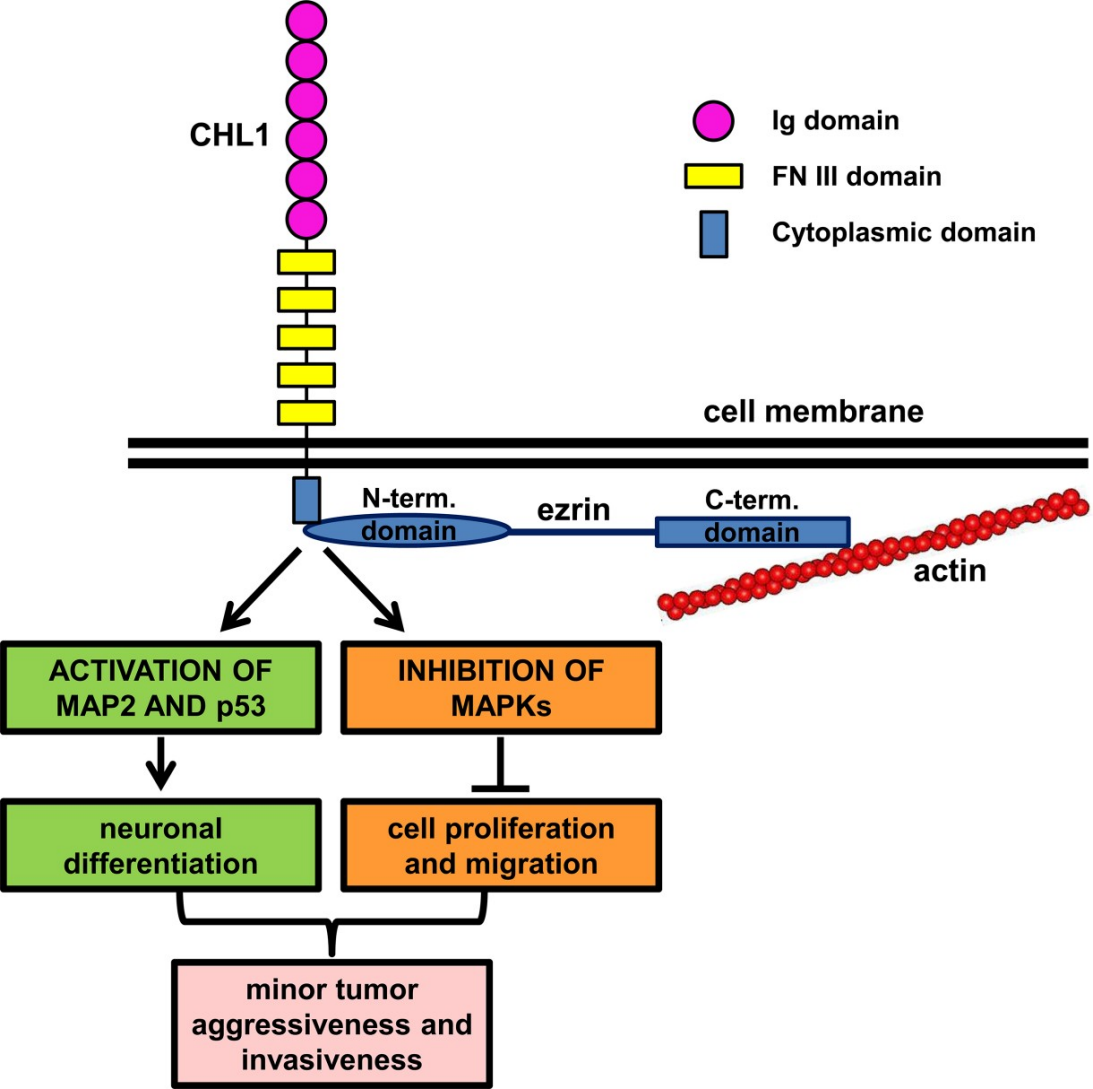
Schematic model of proposed CHL1-ezrin signaling in NB cells. The intra-cytoplasmic CHL1 C-terminal domain interacts with the N-terminal domain of ezrin, which links cytoskeletal actin through its C-terminal domain. This molecular binding activates MAP2 and p53 pathways and inhibits MAPK phosphorylation, inducing neuronal differentiation rather than cell proliferation and migration, thus conferring less aggressive features on NB (The CHL1 extracellular portion contains six Immunoglobulin (Ig)-like domains and five Fibronectin (FN) type III domains).

The reason why ezrin is so important in neuronal differentiation is not well known, yet. Ezrin is involved in many signaling pathways, in particular in the ones including MAPKs and small GTP-ase proteins [26, 27], and it has also been proposed as mediator of neuritogenesis by inactivation of RhoA [11] and activation of Ras [28]. Ezrin, like all ERM proteins, has a C-terminal domain, which binds to actin, and an N-terminal domain, which binds to protein targets of the plasma membrane, where these molecules receive external signals. The two domains are linked together when ezrin is in its inactive conformation and therefore only when ezrin is activated by the phosphorylation of specific sites, they can interact with cytoskeleton and membrane proteins [29]. Ezrin can thus bind to the CHL1 cytoplasmic domain, through the membrane proximal motif described by Schlatter *et al*. [8]. This linkage is likely to trigger differentiation pathways, such as MAP2 phosphorylation, which is a crucial step towards neuronal cytoskeleton development [30], and the activation of the tumor suppressor p53 [31]. At the same time, MAPKs pathways, normally activated by ezrin [32], turned out to be hampered (Fig 6). Ezrin is mostly known as a molecule correlated with tumor progression [33], able to provoke EMT transition and metastases [34, 35], also when it interacts with L1 cell adhesion molecules [36]. Nevertheless, it can play a different role in specific malignancies, such as in urothelial cancer, where high ezrin expression is associated with better survival rates [37, 38], exactly as we found for NB survival, consulting public patients’ datasets. Very few data are available on ezrin role in NB differentiation degree, except for data on some specific NB cell lines that preferentially expressed ezrin if induced to differentiate [12], while nothing is known about ezrin influence on NB aggressiveness. Interestingly, *EZRIN* gene is located at chromosome 6q25.3 and the loss of the distal chromosome 6q has been recently identified as a marker of very poor survival in high-risk NB patients [39, 40], which supports the hypothesis that the lack of ezrin might be detrimental for NB outcome.

In conclusion, our data identified ezrin as a determining effector molecule that cooperates with the NB tumor suppressor CHL1 in inducing neuronal differentiation and lower tumor aggressiveness. Since cell differentiation is considered one of the possible ways to contain cancer propagation, ezrin appears a promising target for the treatment of NB.

## Supporting information

S1 Fig *CHL1* gene expression and NB stage. **(A)** Using the Versteeg NB patients’ dataset from the R2 Genomics Analysis and Visualization Platform (http://r2.amc.nl), patients were divided into high (blue) and low (red) *CHL1* gene expression groups by median-centered Log_2_ ratios. Event-free survival curves were generated for patients with disease stage 1, 2, 3 and 4S (left) or with stage 4 (right). **(B)** Using the SEQC NB patients’ dataset, relative *CHL1* expression levels were plotted in patients with disease stage (st) 1, 2, 3, 4 and 4S, respectively. Patients’ numbers (n) are shown in parentheses.
(TIF)

S2 Fig *CHL1* gene expression and age at diagnosis or *MYCN* status. Using the Versteeg or SEQC NB patients’ datasets in the R2 Genomics Analysis and Visualization Platform, patients were divided into high (blue) and low (red) *CHL1* gene expression groups. Relapse-free or event-free survival (top) and overall survival (bottom) curves were generated for **(A)** patients aged ≤18 months at diagnosis (left) or aged >18 months at diagnosis (right), and **(B)** patients with *MYCN* single copy tumors (left) or with *MYCN* amplified tumors (right). Patients’ numbers (n) are shown in parentheses.
(TIF)

S3 Fig *CHL1* gene expression and chromosomes 1p, 11q, and 17q status. Using the R2 Genomics Analysis and Visualization Platform, in the Lastowska (left) and Maris (right) datasets, *CHL1* gene expression was compared in patients with **(A)** wild-type (WT) chromosome 1p or with 1p deletion (Del), **(B)** wild-type chromosome 11q or with 11q deletion, and **(C)** wild-type chromosome 17q or with 17q gain. Patients’ numbers (n) are shown in parentheses.
(TIF)

S4 Fig *EZRIN* gene expression and chromosomes 1p, 11q, and 17q status. Using the R2 Genomics Analysis and Visualization Platform, in the Lastowska (left) and Maris (right) datasets, *EZRIN* gene expression was compared in patients with **(A)** wild-type (WT) chromosome 1p or with 1p deletion (Del), **(B)** wild-type chromosome 11q or with 11q deletion, and **(C)** wild-type chromosome 17q or with 17q gain. Patients’ numbers (n) are shown in parentheses.
(TIF)

S1 Raw images Original western blot images. (PDF)
