# Expression of Concern: Ezrin interacts with the tumor suppressor CHL1 and promotes neuronal differentiation of human neuroblastoma

**DOI:** 10.1371/journal.pone.0345070

**Published:** 2026-03-17

**Authors:** The PLOS One Editors

Contrary to the Data Availability statement in [1], the original raw quantitative data files supporting the results of [1] were not provided with the article. At editorial request the corresponding author provided the mean and standard deviation values underlying Figs 2F, 3B, 3D-E, 4B-C, 4E-F, and 5A-D (S1 File), and supporting data for Figs 1A-B (S2-S3 Files), but stated that the individual-level data underlying Figs 2-5 and S1-S4 are no longer available.

As the original raw quantitative data files supporting all the results of [1] have not been made available, the article does not comply with the PLOS Data Availability policy, and the *PLOS One* Editors issue this Expression of Concern.

After the publication of [1], a concern was raised that lanes 3 and 5 in Fig 2G appear similar. Given that the underlying western blot for Fig 2G was provided with [1] in S1 Raw images, PLOS considers this concern resolved.

## Supporting information

S1 File Underlying quantitative data (means and standard deviations only) in support of Figs 2–5. (XLSX)

S2 File The file obtained by the SEQC patients’ datasets as described in Fig 1A. (PPTX)

S3 File The file obtained by the SEQC patients’ datasets as described in Fig 1B. (PPTX)
