# Supplementary figures and images for: Ezrin interacts with the tumor suppressor CHL1 and promotes neuronal differentiation of human neuroblastoma
Source: PLoS One. 2020 Dec 16;15(12):e0244069. doi: 10.1371/journal.pone.0244069 (PMC7743987; doi:10.1371/journal.pone.0244069)

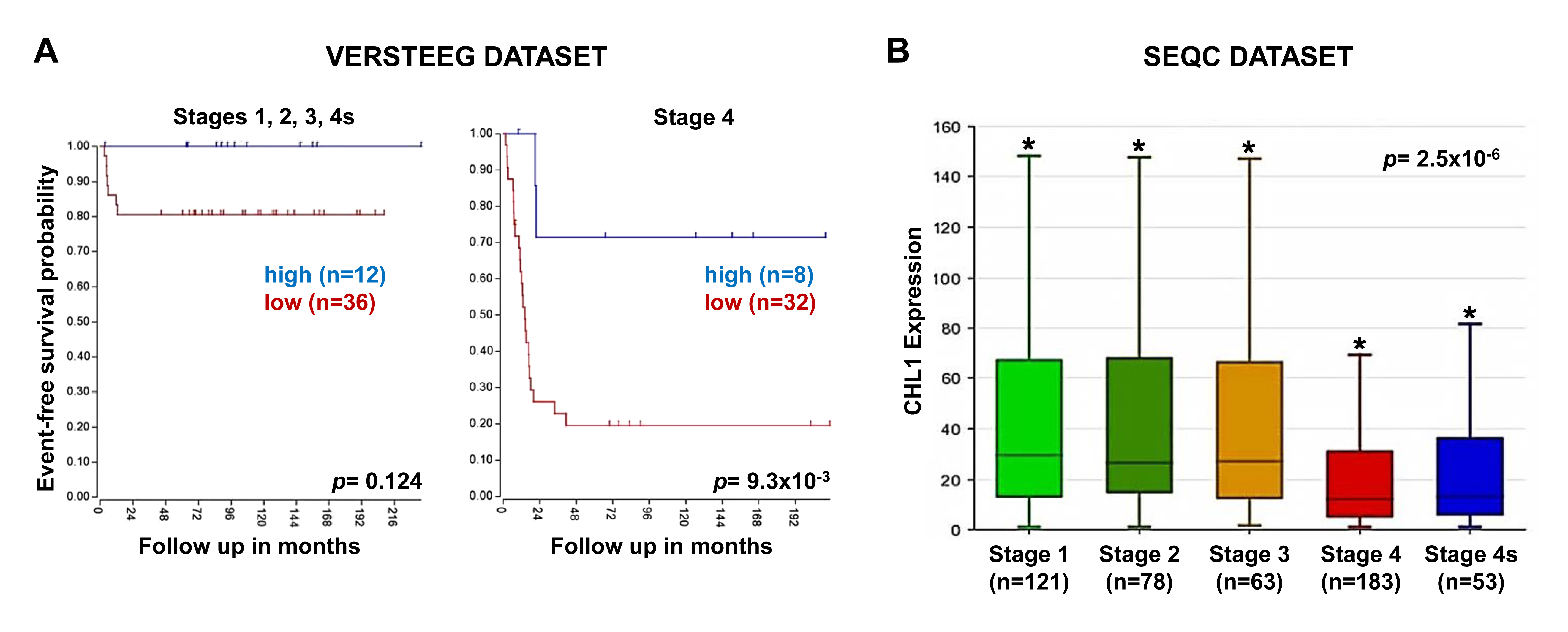

Supplement: S1 Fig — (A) Using the Versteeg NB patients’ dataset from the R2 Genomics Analysis and Visualization Platform (http://r2.amc.nl), patients were divided into high (blue) and low (red) CHL1 gene expression groups by median-centered Log2 ratios. Event-free survival curves were generated for patients with disease stage 1, 2, 3 and 4S (left) or with stage 4 (right). (B) Using the SEQC NB patients’ dataset, relative CHL1 expression levels were plotted in patients with disease stage (st) 1, 2, 3, 4 and 4S, respectively. Patients’ numbers (n) are shown in parentheses. (TIF) [file pone.0244069.s001.tif]

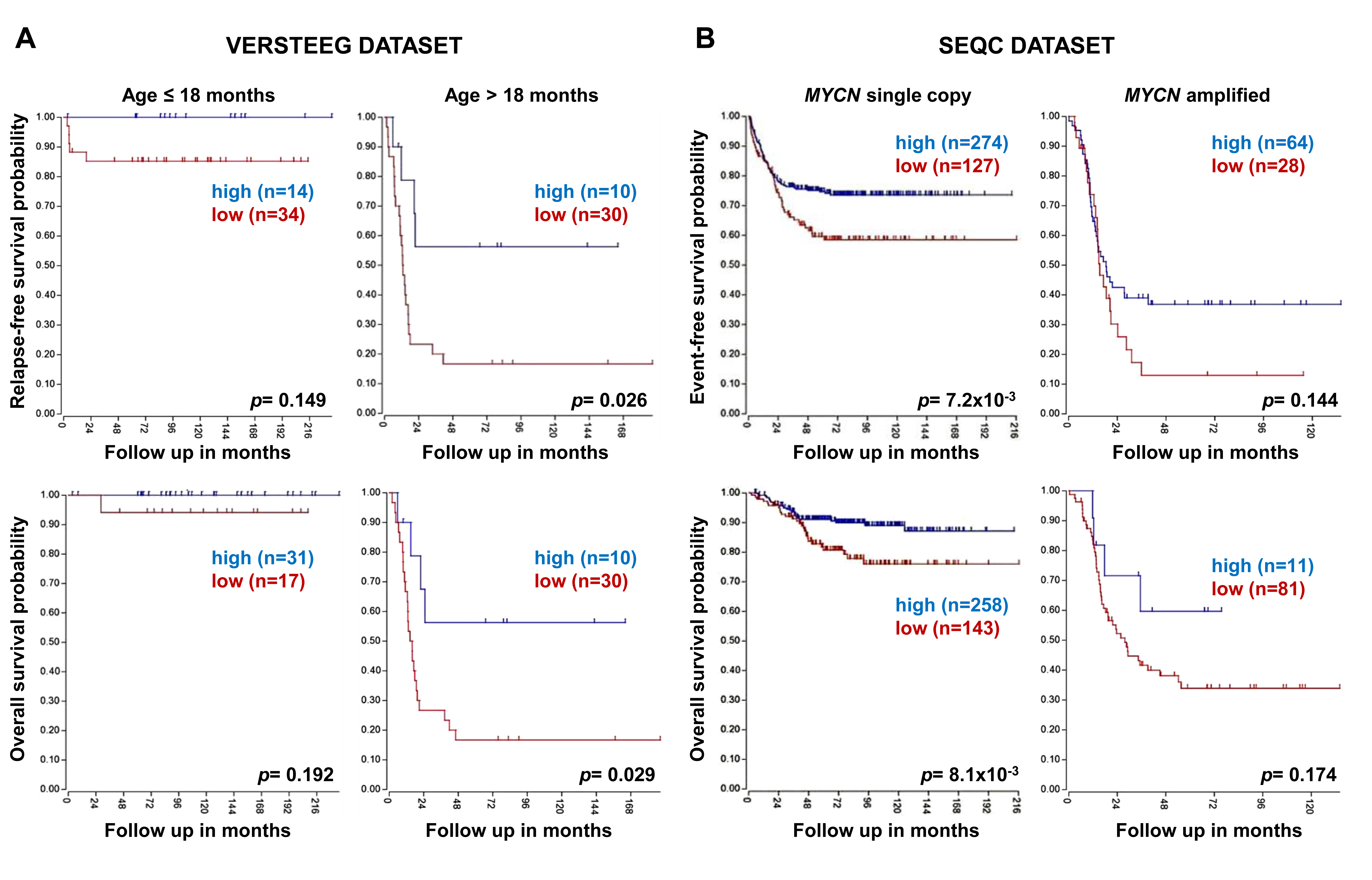

Supplement: S2 Fig — Using the Versteeg or SEQC NB patients’ datasets in the R2 Genomics Analysis and Visualization Platform, patients were divided into high (blue) and low (red) CHL1 gene expression groups. Relapse-free or event-free survival (top) and overall survival (bottom) curves were generated for (A) patients aged ≤18 months at diagnosis (left) or aged >18 months at diagnosis (right), and (B) patients with MYCN single copy tumors (left) or with MYCN amplified tumors (right). Patients’ numbers (n) are shown in parentheses. (TIF) [file pone.0244069.s002.tif]

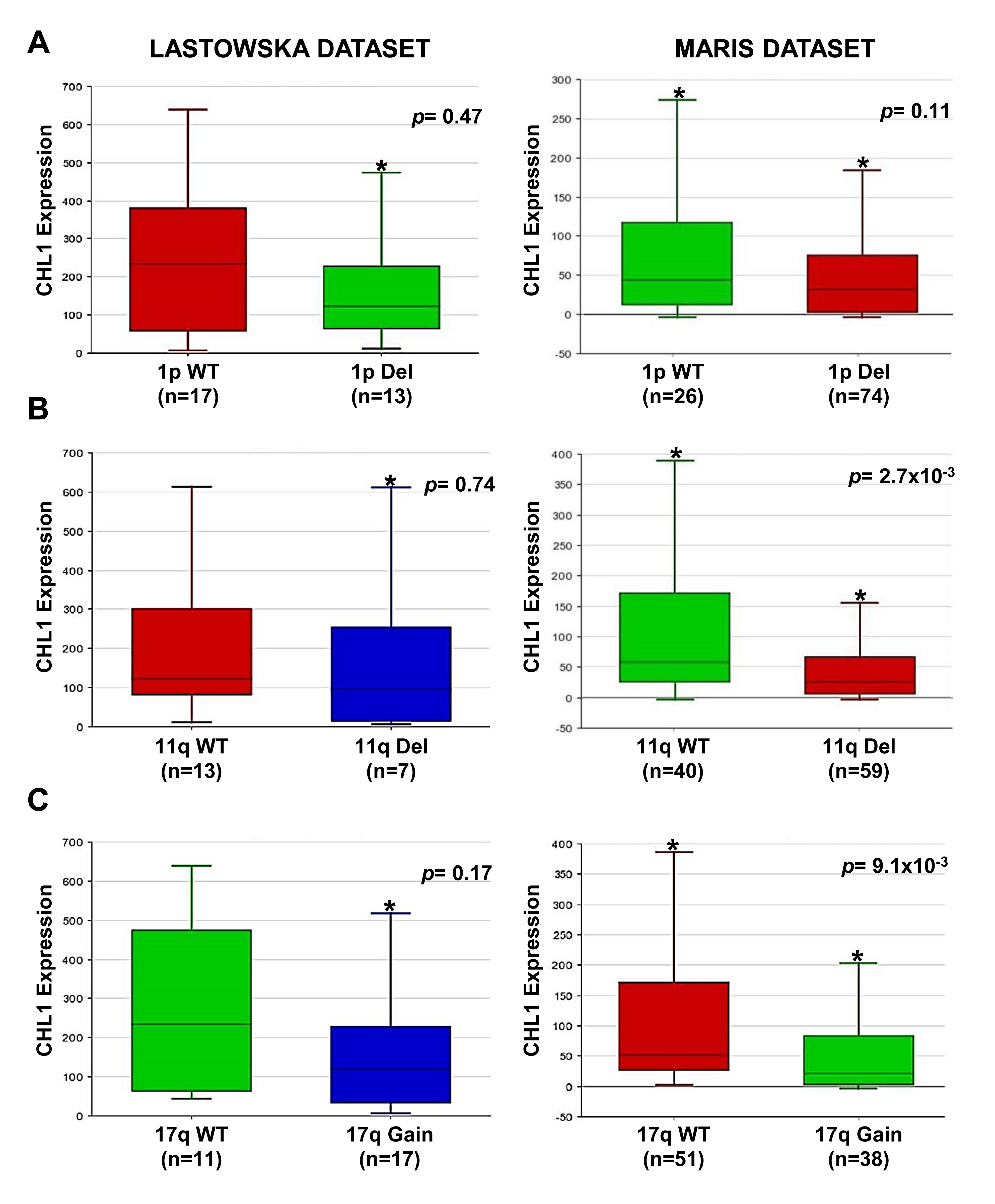

Supplement: S3 Fig — Using the R2 Genomics Analysis and Visualization Platform, in the Lastowska (left) and Maris (right) datasets, CHL1 gene expression was compared in patients with (A) wild-type (WT) chromosome 1p or with 1p deletion (Del), (B) wild-type chromosome 11q or with 11q deletion, and (C) wild-type chromosome 17q or with 17q gain. Patients’ numbers (n) are shown in parentheses. (TIF) [file pone.0244069.s003.tif]

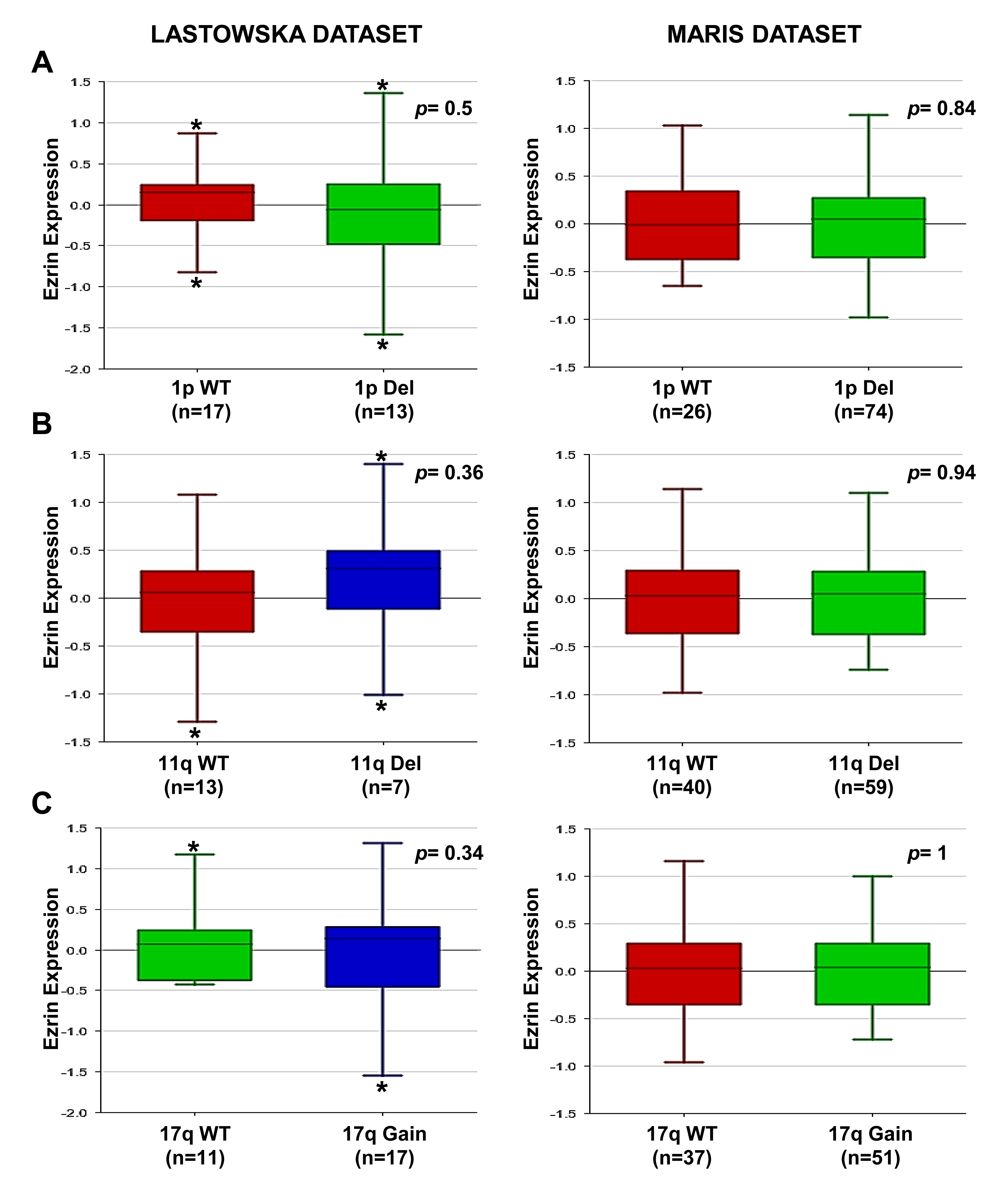

Supplement: S4 Fig — Using the R2 Genomics Analysis and Visualization Platform, in the Lastowska (left) and Maris (right) datasets, EZRIN gene expression was compared in patients with (A) wild-type (WT) chromosome 1p or with 1p deletion (Del), (B) wild-type chromosome 11q or with 11q deletion, and (C) wild-type chromosome 17q or with 17q gain. Patients’ numbers (n) are shown in parentheses. (TIF) [file pone.0244069.s004.tif]
