# Supplementary material for: Ezrin interacts with the tumor suppressor CHL1 and promotes neuronal differentiation of human neuroblastoma
Source: PLoS One. 2020 Dec 16;15(12):e0244069. doi: 10.1371/journal.pone.0244069 (PMC7743987; doi:10.1371/journal.pone.0244069)

Ezrin interacts with the tumor suppressor CHL1 and promotes neuronal differentiation of human neuroblastoma

Marzia Ognibene and Annalisa Pezzolo

Laboratorio Cellule Staminali Post Natali e Terapie Cellulari, IRCCS Istituto Giannina Gaslini, Genova, 16147, Italy.

Statement about the original images for the blots presented in the manuscript

We have collected all the original images for blots in a PowerPoint file before building a single PDF file, because we needed to show a large number of images and, in that way we could obtain a more easily manageable file. Beside every group of original images we have put the corresponding figure of the manuscript, to immediately check each blot. We can assure that the presented images in the PDF file have not lost in resolution.

Since images were all obtained with a Bio-Rad ChemiDoc instrument, sometimes the area of interest was directly cropped and saved, if nothing else specific or peculiar was detectable on the blot.

When possible, we stripped the membranes and we probed them again with other antibodies, or, alternatively, we cut one membrane at different levels of the protein marker, in order to obtain different fragments to probe with different antibodies at the same time. Consequently, for each Western blot panel there is only one image for the anti-actin antibody, or we have chosen one anti-actin re-probing representative for all the blots in the panel.

We use a pre-stained protein marker that leaves very faint bands, and it is undetectable in chemiluminescence, so we have to retrace it every time on the membrane with a pencil. After evaluating the right bands merging the colorimetric image containing the protein ladder with the chemiluminescence image, we usually save the best images to be used for the manuscript without maintaining the retraced markers on them. We have indicated in every figure the molecular weight of the detected proteins.

We did not include positive controls in the blots because we probed them with antibodies for which we already knew the specific pattern for the NB cell lines here used.

We performed three independent experiments for each western-blot figure, and every blot in the figure came from the same experiment, therefore from the same protein lysate for each cell line: the figures prepared for the manuscript show the best experiment among the three.

Uncropped blots for Fig 2C

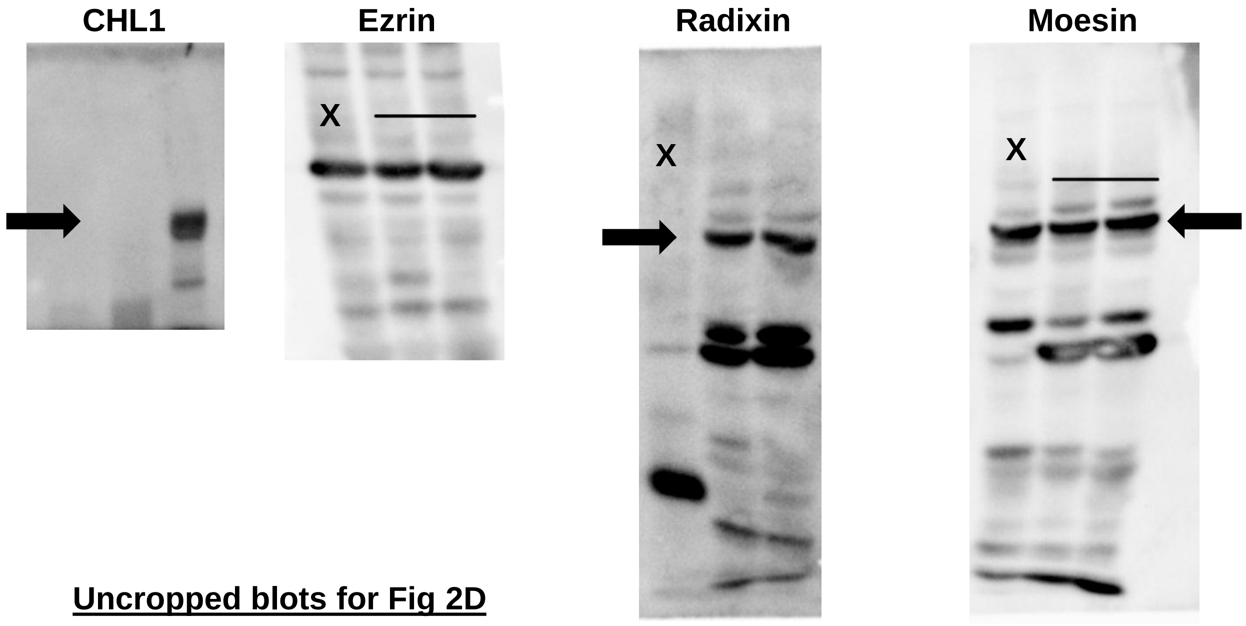

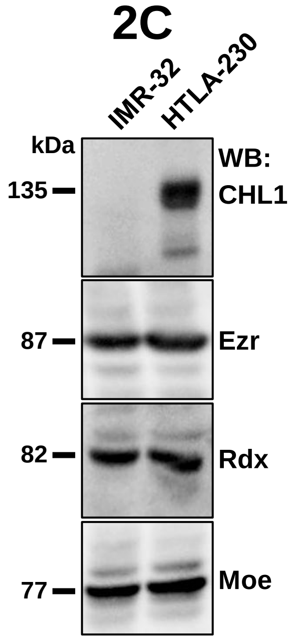

Uncropped blots for Fig 2D

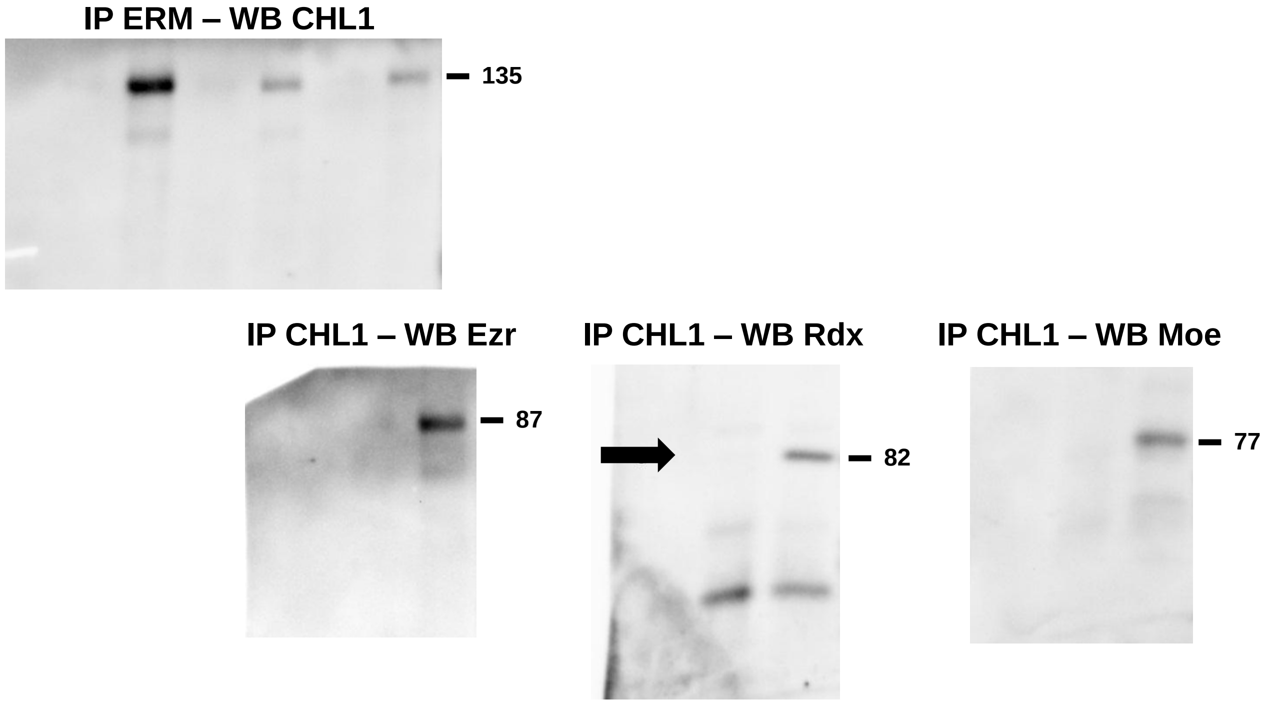

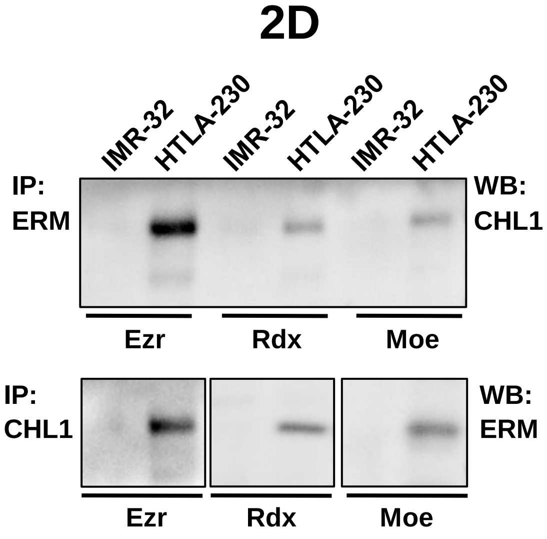

Uncropped blots for Fig 2E

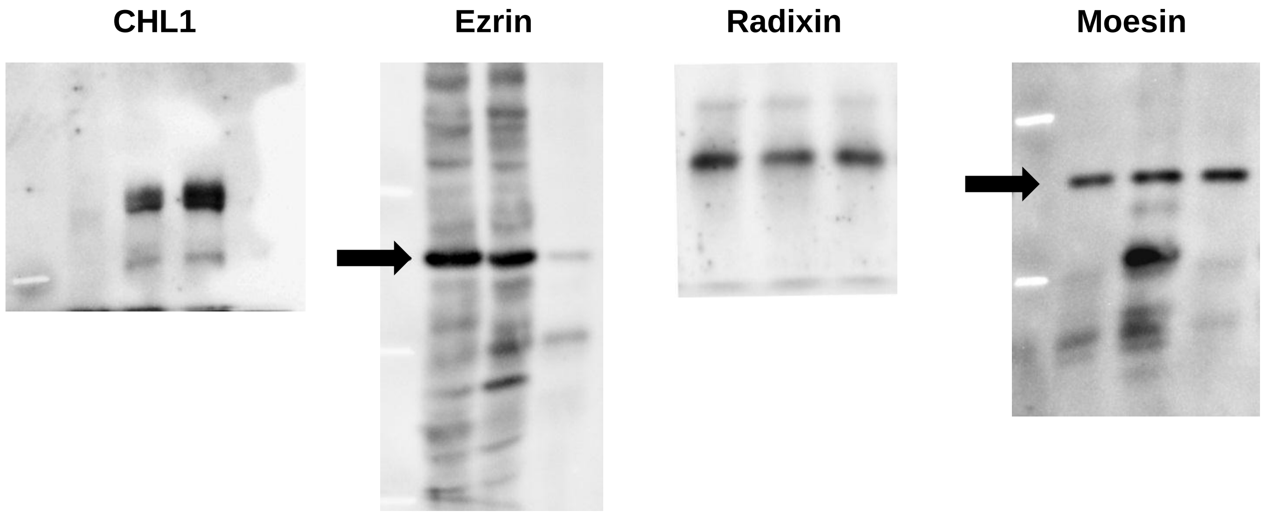

2E

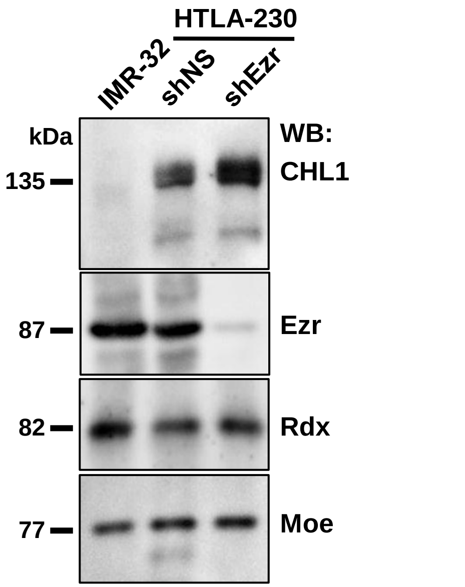

Uncropped blot for Fig 2G

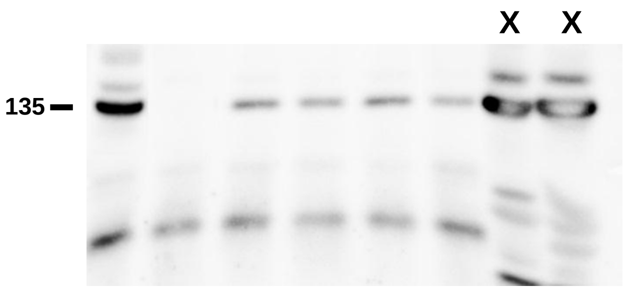

2G

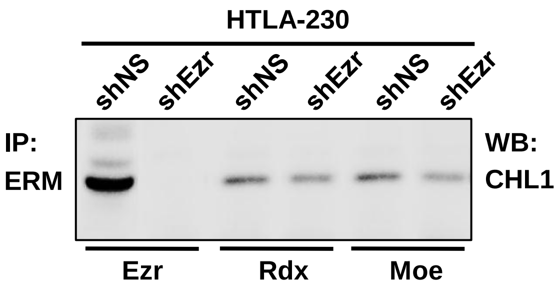

Uncropped blots for Fig 3A

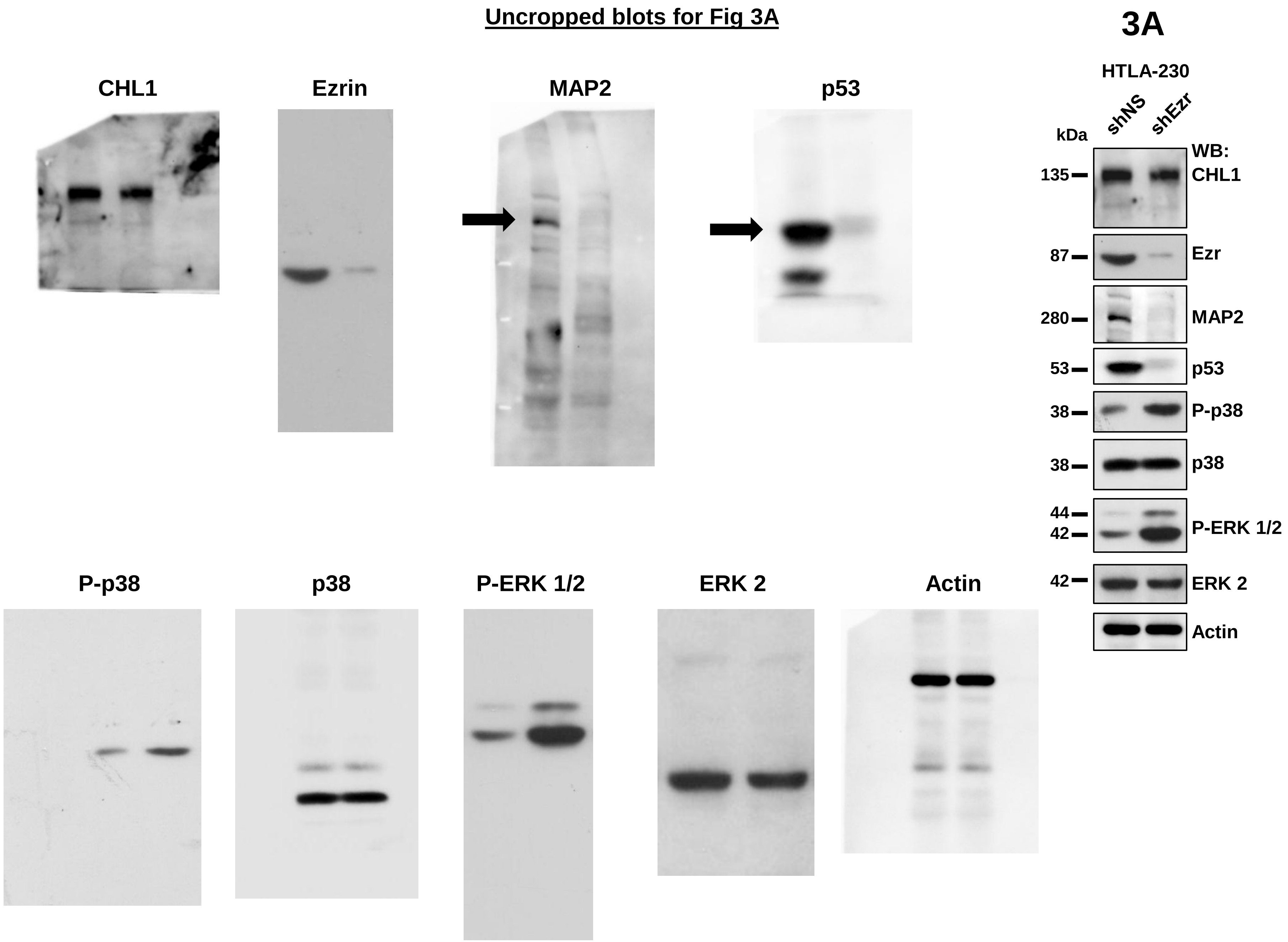

Uncropped blots for Fig 3C

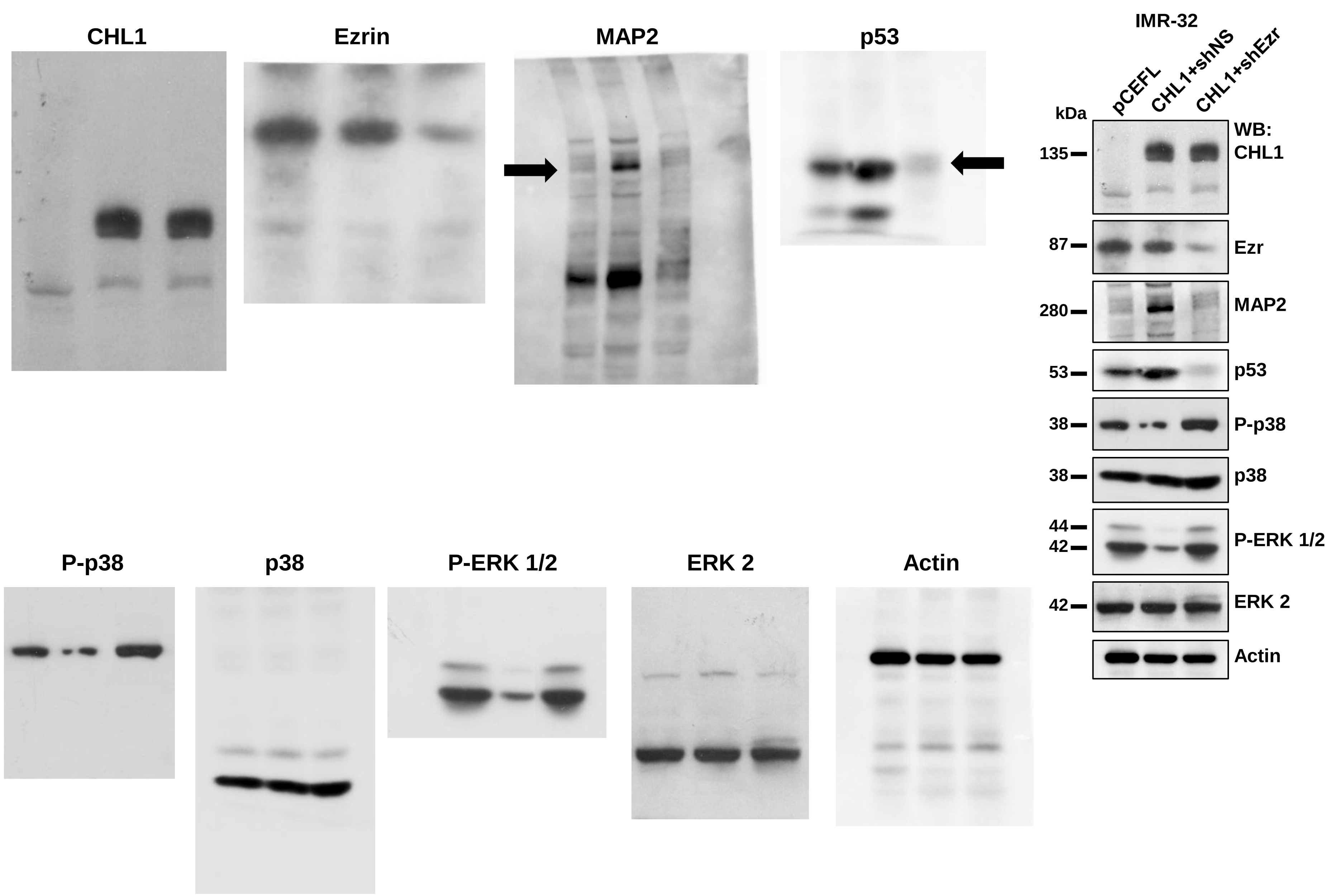

Supplement: S1 Raw images — (PDF) [file pone.0244069.s005.pdf]
