# Supplementary figures and images for: Expression of Concern: Ezrin interacts with the tumor suppressor CHL1 and promotes neuronal differentiation of human neuroblastoma
Source: PLoS One. 2026 Mar 17;21(3):e0345070. doi: 10.1371/journal.pone.0345070 (PMC12994805; doi:10.1371/journal.pone.0345070)

## Slide 1
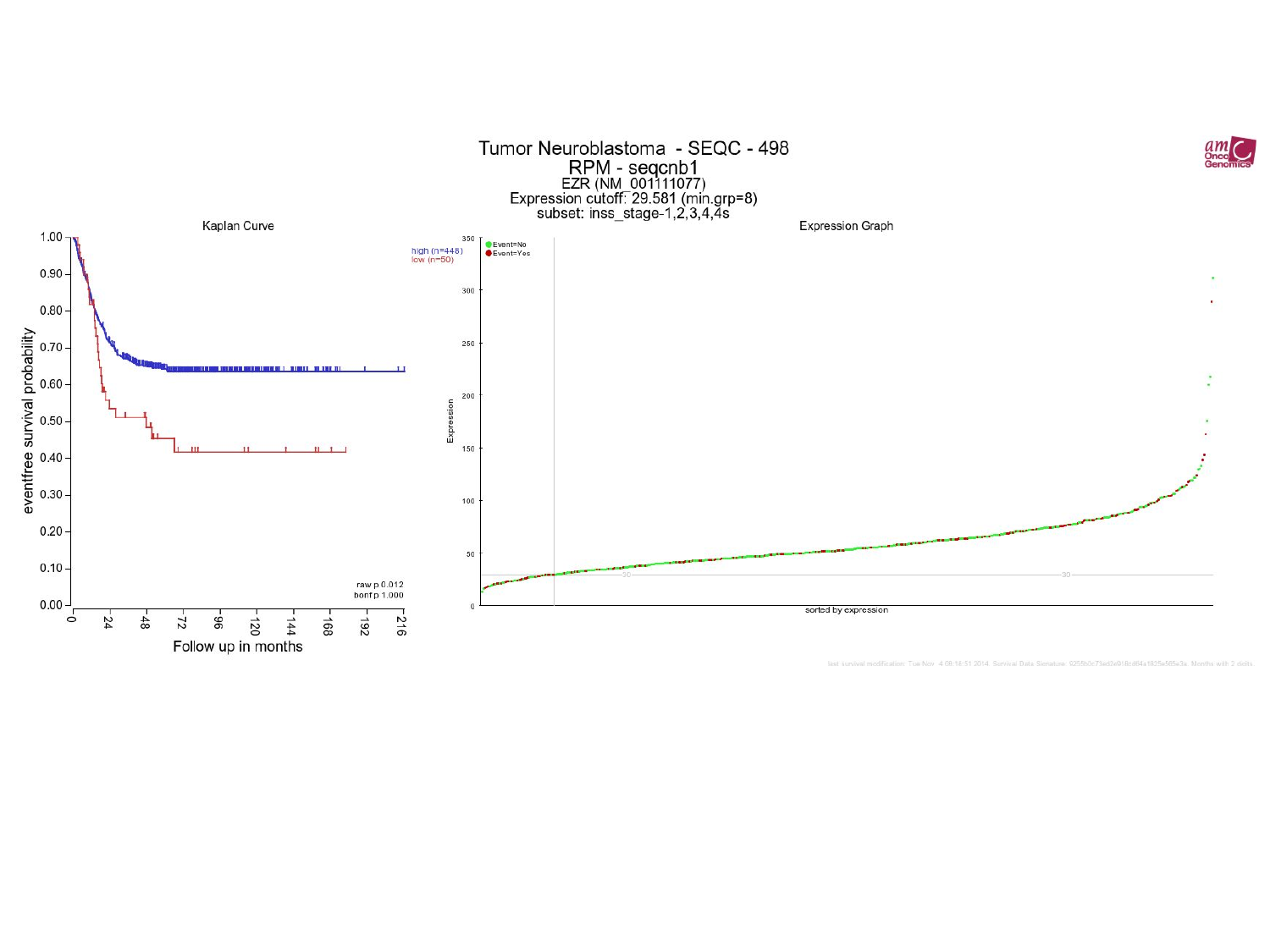

## Slide 2
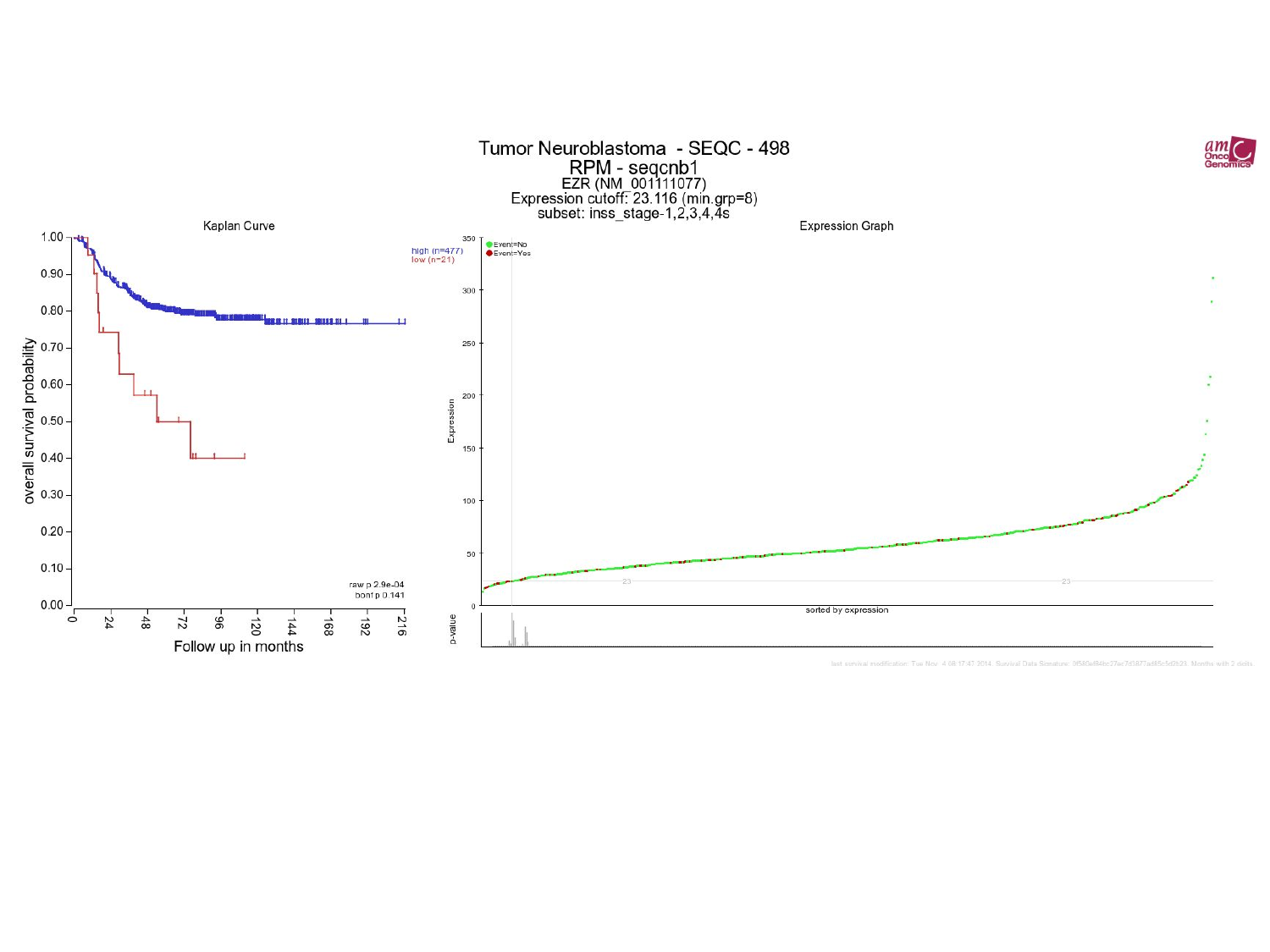

Supplement: S2 File — (PPTX) [file pone.0345070.s002.pptx]

## Slide 1
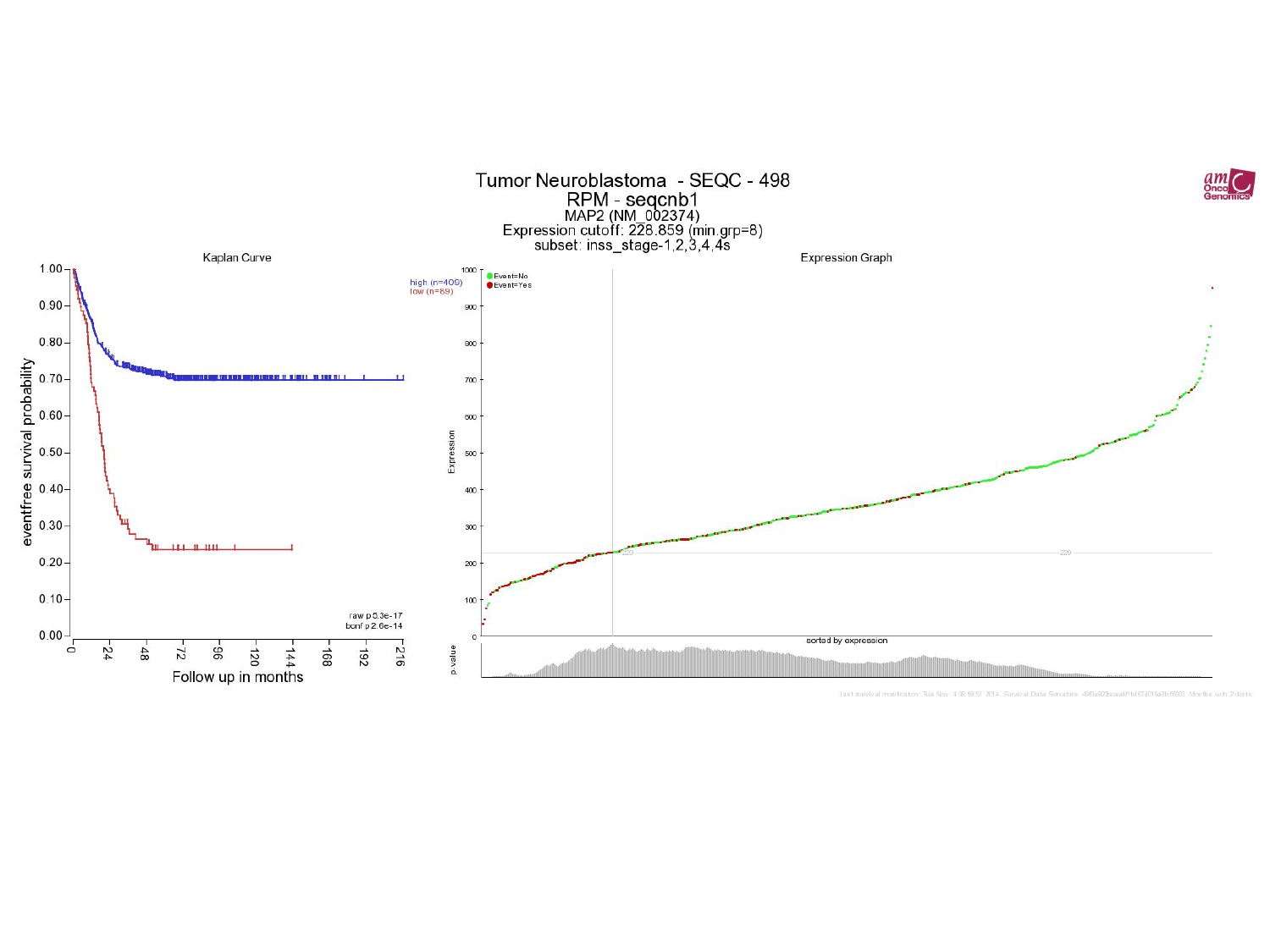

## Slide 2
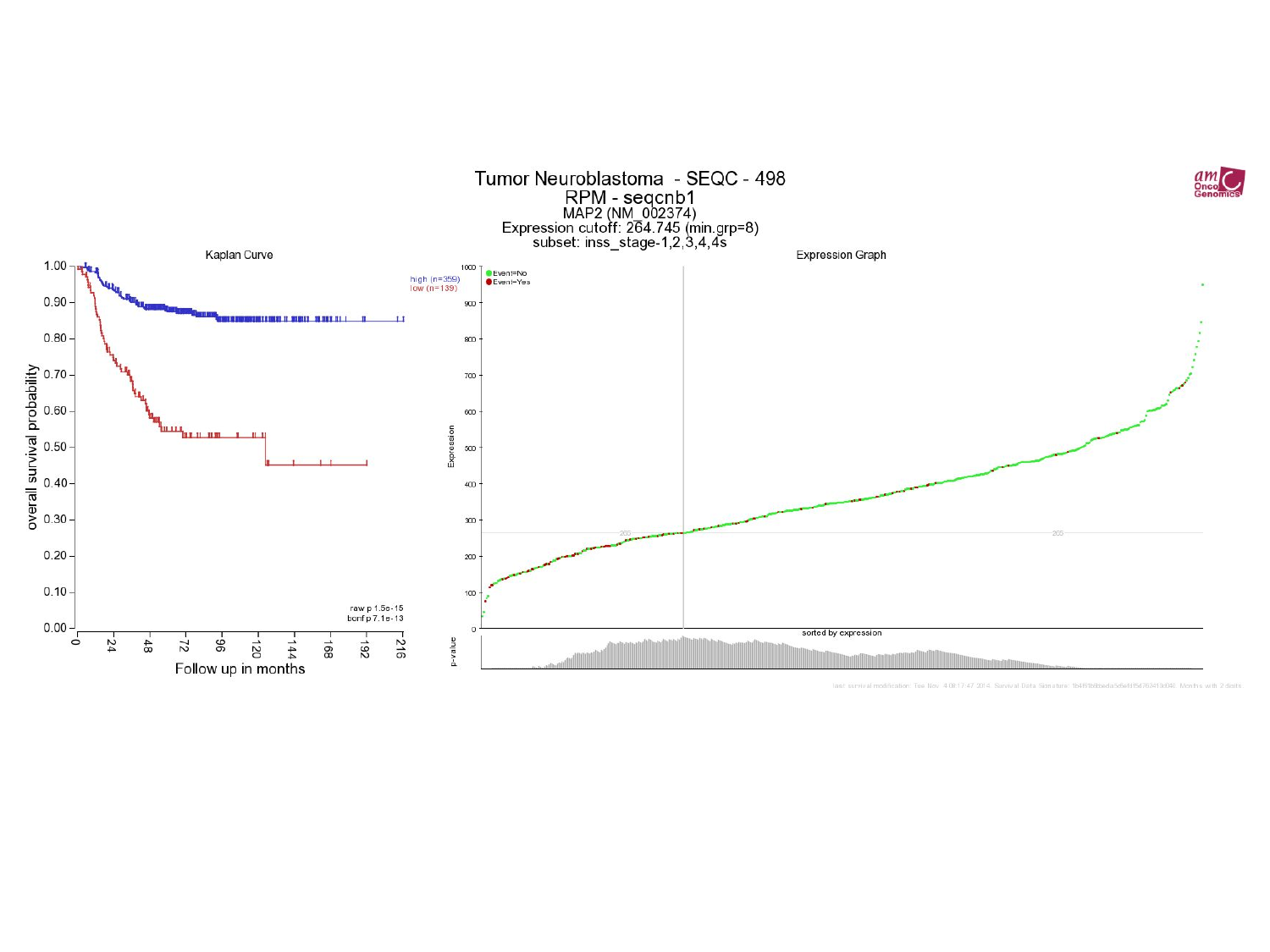

Supplement: S3 File — (PPTX) [file pone.0345070.s003.pptx]
